# FishChip: Development of a High‐Throughput qPCR Assay for Freshwater Fish eDNA Quantification and Its Comparison With Droplet Digital PCR

**DOI:** 10.1111/1755-0998.70187

**Published:** 2026-08-05

**Authors:** Shoutao Cheng, Jiasheng Zhang, Zhuming Liang, Hao Xue, Lingsong Zhang, Yeyao Wang, Fansheng Meng

**Affiliations:** ^1^ State Key Laboratory of Environmental Criteria and Risk Assessment, Chinese Research Academy of Environmental Sciences Beijing People's Republic of China; ^2^ Key Laboratory for National Park Protection and Development of Hainan Province/Hainan Institute of National Park Haikou People's Republic of China; ^3^ China National Environmental Monitoring Center Beijing People's Republic of China

**Keywords:** aquatic, droplet digital PCR, environmental DNA, fish, high‐throughput qPCR assay

## Abstract

Quantitative monitoring of fish using environmental DNA (eDNA) typically relies on quantitative real‐time PCR (qPCR) and droplet digital PCR (ddPCR), yet both methods are constrained in throughput, making it difficult to meet the demands of basin‐scale surveys. High‐throughput qPCR (HT‐qPCR) has emerged as a platform capable of simultaneously processing large numbers of targets and samples, but its performance for fish eDNA quantification and its comparability with ddPCR remain poorly characterized. Here, we selected nine widely distributed freshwater fishes and designed 19 species‐specific primer pairs (2–3 per species). All assays performed well on a high‐throughput chip‐based qPCR (HT‐qPCR) platform, with amplification efficiencies ranging from 91.07% to 105.83% and 78.9% of assays falling within the optimal 95%–105% interval. Applying HT‐qPCR to quantify fish eDNA across six aquatic regions, *Cyprinus carpio* ( *C. carpio* ) achieved a 100% detection rate. For the same fish species, the two primer sets exhibited measurable differences in detection rate and quantitative estimates. HT‐qPCR and ddPCR estimates were strongly correlated (*r* = 0.858), and Deming regression indicated near‐equivalence (HT‐qPCR = 0.52 + 0.99 × ddPCR). Bland–Altman analysis revealed a consistent ~3‐fold overestimation by HT‐qPCR relative to ddPCR, which is generally considered the more accurate method, suggesting that a correction factor could harmonize data across platforms. These results validate the utility of HT‐qPCR for catchment‐scale quantification of fish eDNA concentrations and provide methodological innovation and technical support for aquatic ecosystem monitoring and assessment.

## 1 Introduction

Fish communities serve as critical biological indicators for aquatic ecosystem health assessment, deriving their ecological indicative value from distinctive morphological features and widespread distribution (Li et al. 2018), occupation of upper trophic levels in aquatic food chains (Zhang et al. 2019), multidimensional sensitivity to environmental stressors (Wang et al. 2021), and their role in maintaining ecosystem stability (Villéger et al. 2017). Traditional fish monitoring methods (such as gill netting, trawling, and electrofishing) present significant limitations including high operational costs, dependence on professional taxonomic teams (Nygård et al. 2016), and potential physical disturbance to fish populations and their habitats (Hering et al. 2018). In contrast, environmental DNA (eDNA) technology captures genetic material shed by organisms in water bodies, enabling simultaneous detection and precise identification of multiple species (Taberlet et al. 2012). Its advantages primarily manifest as non‐invasive sampling, high sensitivity, cost‐effectiveness, and shortened procedural timeframes (Wang et al. 2021), factors that have led to its incorporation into the official monitoring framework of the European Union Water Framework Directive (Hering et al. 2018).

eDNA technology has demonstrated exceptional application potential in aquatic ecosystem monitoring, successfully encompassing the full spectrum of aquatic habitats (Rees et al. 2014), including rivers (Gao et al. 2023), lakes (He et al. 2022), streams (Doi et al. 2017), estuaries (Gibson et al. 2024), and marine (Stoeckle et al. 2021) environments. From a research depth perspective, eDNA technology has evolved from simple “species presence confirmation” to “ecological function analysis”, establishing a comprehensive research framework including species monitoring, population dynamics estimation, community structure analysis, and functional diversity assessment (Marques et al. 2021). Research has confirmed that eDNA concentration in water bodies exhibits a significant positive correlation with species abundance and biomass (Mizumoto et al. 2018), providing a theoretical foundation for the application of eDNA technology in quantitative monitoring of fish resources. However, a meta‐analysis found that these correlations are generally weaker under natural conditions than in the laboratory (Yates et al. 2019), as eDNA concentration can be influenced by various biological and technical factors. Despite these influences, quantitative eDNA data remain ecologically meaningful and can provide valuable insights when interpreted with appropriate caution. Species‐specific detection primarily employs methods including real‐time quantitative PCR (qPCR), multiple qPCR, quantitative MiSeq (qMiSeq), and droplet digital PCR (ddPCR), enabling precise measurement of fish eDNA concentrations and scientific estimation of fish biomass and population abundance (Wang et al. 2021). qPCR, as the current mainstream detection method, is limited to one species per reaction, requires greater DNA sample consumption as the number of target species increases, and is susceptible to inhibition by organic substances in environmental samples (Doi, Takahara, et al. 2015). Although multiplex qPCR can simultaneously detect two to five species per reaction, it still cannot meet the demands of large‐scale, multi‐species monitoring (Tsuji et al. 2018). Compared to qPCR, ddPCR offers improved resistance to PCR inhibitors (Dingle et al. 2013), higher sensitivity for low‐abundance species, and does not require a standard curve, making it more accurate and operationally simpler (Doi, Takahara, et al. 2015). ddPCR does not resolve the throughput constraints inherent to conventional qPCR. Each reaction accommodates one or two target markers, and instrument capacity limits the number of samples and species that can be processed per run. High equipment and reagent costs (Hunter et al. 2017) compound this limitation, restricting the utility of ddPCR in monitoring programs that require simultaneous quantification of numerous species across large sample sets. More recently, CRISPR‐based approaches have also been applied to eDNA species detection (Williams et al. 2019), offering rapid, field‐deployable identification (Wei et al. 2023), though their quantitative capabilities remain limited. However, these approaches are designed for single‐species workflows and are not suited for simultaneous multi‐species quantification across large sample sets.

High‐throughput quantitative PCR (HT‐qPCR), also referred to as chip‐based qPCR or chip qPCR, uses microfluidic chip technology to perform thousands of nanoliter‐scale PCR reactions within a single experimental run. Conventional qPCR is restricted to a small number of targets per reaction and requires relatively large reagent volumes; HT‐qPCR resolves both constraints by quantifying hundreds of target genes across multiple samples simultaneously, substantially increasing throughput while reducing per‐reaction reagent consumption (Waseem et al. 2019). The nanoliter‐scale volumes inherent to microfluidic chips dilute inhibitory substances present in environmental samples, which can improve detection sensitivity in complex matrices where conventional qPCR performance is compromised. Researchers demonstrated the feasibility of this approach using larvae of 23 commercially valuable bivalve and crustacean species: 42 detection methods were selected and validated, genetic signals correlated closely with direct microscopic counts, and species‐level quantification was achieved with substantially improved time and cost efficiency compared to conventional methods (van der Pouw Kraan et al. 2024).

Currently, systematic comparative studies of HT‐qPCR versus ddPCR in fish eDNA detection remain insufficient. This research aims to: (1) screen and design species‐specific primers applicable to nine widely distributed freshwater fish species occupying different trophic levels; (2) develop a HT‐qPCR‐based assay, termed FishChip, for simultaneous quantification of multiple freshwater fish species from environmental DNA samples; (3) evaluate and compare the performance of HT‐qPCR and ddPCR in eDNA quantitative detection, validating the method's applicability across different geographical regions of freshwater systems.

## 2 Materials and Methods

### 2.1 Study Sites and Water Sampling

**FIGURE 1:**
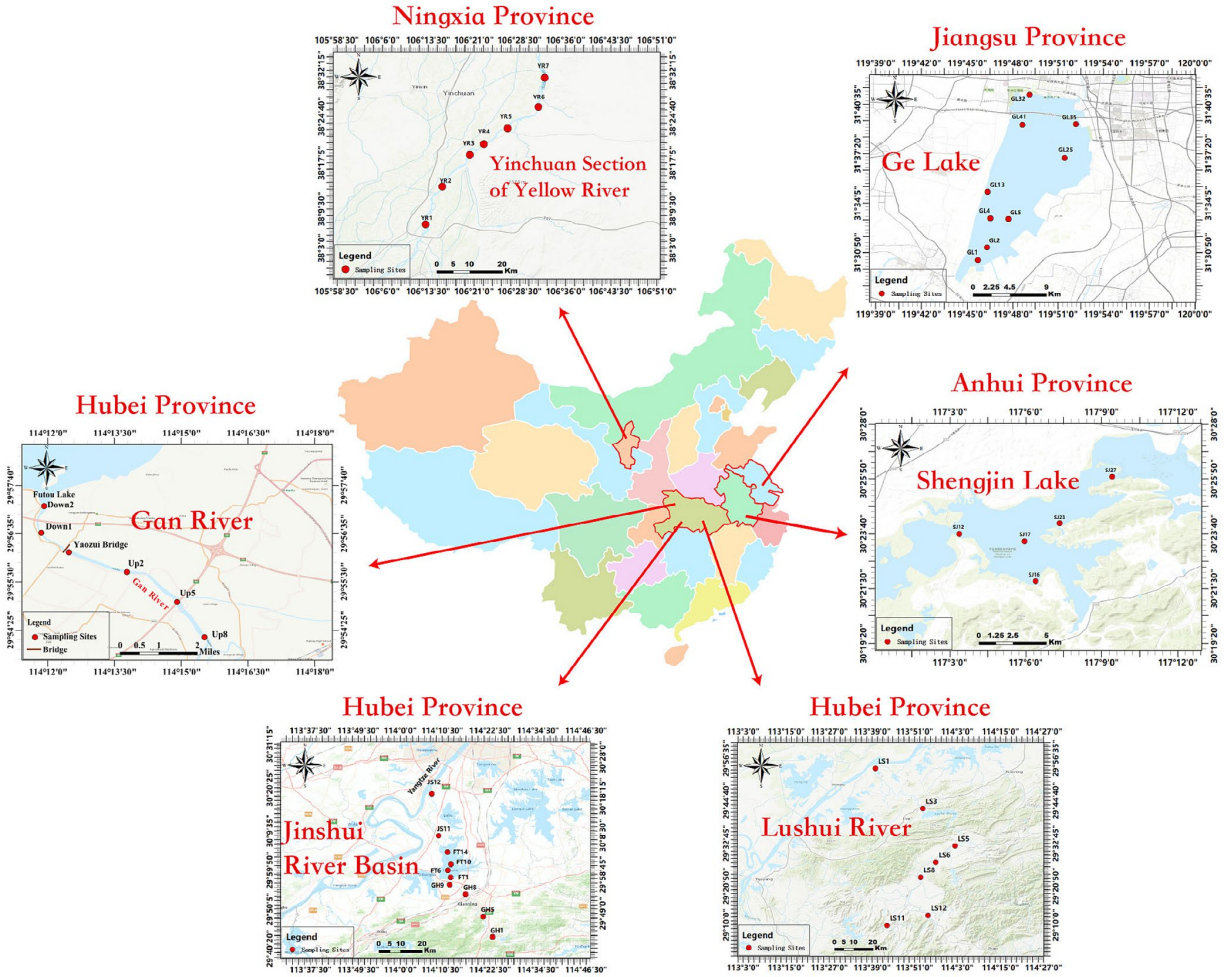
Geographic distribution of eDNA sampling sites across six water bodies in four provinces of China, including the Yinchuan Section of the Yellow River (Ningxia Province), Ge Lake (Jiangsu Province), Shengjin Lake (Anhui Province), Gan River, Jinshui River Basin, and Lushui River (Hubei Province). Red dots indicate sampling locations.

A total of 44 sampling sites were established across 6 geographical regions in China, encompassing two aquatic ecosystem types: 3 rivers and 2 lakes (Figure 1). For rivers, sampling included 7 sites on the Yellow River Yinchuan Section in Ningxia (YR), 10 sites in the Jinshui River Basin (JS), 7 sites on the Lushui River (LS), and 6 sites on the Gan River in Hubei (GH). For lakes, sampling included 9 sites on Ge Lake in Jiangsu (GL) and 5 sites on Shengjin Lake in Anhui (SJ). These sites were selected to evaluate the applicability of FishChip across different aquatic ecosystem types and geographic regions in China. Detailed information regarding sampling times and sampling sites can be found in Text S1 and Table S1. The map was created using ArcGis 10.8.1 (ESRI 2020), with the basemap sourced from Esri World Topographic Map via ArcGIS Online. Photographs of field surveys across six sampling regions are displayed in Figure S1.

Water samples were collected at 0.5 m depth using a 5 L plexiglass sampler (PSC‐1A, Changzhou Pun Sen Electronic Instrument Factory, China) to obtain 6 L per site, homogenized and divided into three 2 L sterile bottles as replicates. Between sites, sampling equipment was disinfected with 10% sodium hypochlorite for 30 min and rinsed thrice. Samples were stored at 4°C, filtered within 24 h, and eDNA filter membranes preserved at −20°C until extraction.

### 2.2 eDNA Extraction and Purification

Each litre of water was filtered with 0.45 μm Mixed Cellulose Ester (MCE) microporous filter membranes (JinTeng, Tianjin, China) in triplicate, with concentrated samples sealed in 5 mL cryopreservation tubes (AXYGEN, Union City, CA, USA) and stored at −80°C before environmental DNA extraction. A filter blank of 1 L DEPC water was used to control for contamination. Prior to extraction, each filter membrane was cut into approximately 2 mm fragments using sterile scissors to enhance DNA release efficiency. eDNA extraction using a modified DNeasy Blood & Tissue Kit (Qiagen, Germany) protocol. The eDNA sampling and extraction procedures were conducted following the protocol established by Zhong et al. (Zhong et al. 2022).

### 2.3 Target Fish Species Selection and Characteristics

Based on the geographical distribution breadth in China, trophic level (trophic level index ≤ 2.8 as “low trophic level”, 2.8 < trophic level index < 3.5 as “medium trophic level”, trophic level index ≥ 3.5 as “high trophic level”), ecological status in China (native, introduced, endemic), and feeding habits (herbivorous, planktivorous, carnivorous, omnivorous), nine representative fish species were ultimately selected as study subjects (Table S2): *Oryzias latipes* ( *O. latipes* ), *Micropterus salmoides* ( *M. salmoides* ), *Oreochromis mossambicus* ( *O. mossambicus* ), *Cyprinus carpio* ( *C. carpio* ), *Schizothorax sinensis* ( *S. sinensis* ), *Danio rerio* ( *D. rerio* ), *Pseudorasbora parva* ( *P. parva* ), *Hypophthalmichthys nobilis* ( *H. nobilis* ), *Silurus meridionalis* ( *S. meridionalis* ). Species spanning different trophic levels were included to provide a broader assessment of the detection performance of FishChip across species with different ecological roles and biological characteristics. The above data were primarily sourced from the FishBase database. To confirm the taxonomic identity of the nine fish specimens purchased online, MiFish primers were used to conduct Sanger sequencing (Text S2), and the corresponding NCBI accession numbers are provided in Table S3.

### 2.4 Species‐Specific Primer Design and Validation

To obtain species‐specific primer information, a systematic literature search was conducted, and the retrieved primers were screened. Primer sequences for seven of the nine fish species were identified from the literature. For the remaining two species for which no published primers were available ( *S. sinensis* and *S. meridionalis* ), species‐specific primers were designed based on mitochondrial genome information (Table S3). For each target species, mitochondrial genome sequences of all available congeneric species retrieved from the NCBI database were included in the alignment analysis to maximize the stringency of species‐specific primer design (Table S4). Considering the impact of primer differences on detection results, 2–3 pairs of primers were selected for experimental validation for each fish species. The primer design rules are detailed in Text S3, and the final list of selected primers is provided in Table S5.

### 2.5 Standard Curve Preparation and Quantification Strategy

Plasmid standards used for the standard curve were constructed via TA cloning from genomic DNA of target species; the detailed procedure, and sequence alignment results (Table S6) are provided in Text S4. The experiment utilized plasmid standards containing fish mitochondrial target genes. The concentration of the standards was measured to calculate copy numbers (Text S5), and a standard curve system was established through 10‐fold serial dilution (10^3^–10^9^copies/μL). The gradient‐diluted plasmid standards and no‐template controls (NTC) were amplified under identical conditions using the Wafergen SmartChip real‐time PCR system, with each concentration point replicated at least three times to ensure data reliability. The Ct values (threshold cycle numbers) corresponding to each standard concentration were collected, and a linear regression analysis was performed with the logarithm of the standard concentration as the x‐axis and the corresponding Ct values as the y‐axis to derive the standard curve equation. The NTC should show no amplification or a Ct value significantly higher than the lowest concentration standard to ensure the accuracy of the experimental results. Additionally, the amplification efficiency (Text S6), specificity (Text S7), and sensitivity (Text S8) were validated.

### 2.6 High‐Throughput qPCR Chip System Development

HT‐qPCR was performed on the Wafergen SmartChip Real‐time PCR system (Takara, Japan). Each chip contained 5184 cells. In this study, each chip was configured in an 80‐sample × 64‐assay format, yielding 5120 reactions per run. The 64 assay positions accommodated the 19 primer pairs in triplicate (57 positions), with the remaining positions reserved for no‐template controls. Each HT‐qPCR reaction well (100 nL) contained 1× LightCycler 480 SYBR Green I Master (Roche Applied Sciences, Indianapolis, Indiana), 1 μg/μL bovine serum albumin (New England Biolaboratories, Beverly, Massachusetts), 0.5 μM of each primer, and diluted plasmid at concentrations ranging from 10^3^ to 10^9^ copies/μL.

The thermal cycling conditions included an initial denaturation at 95°C for 10 min, followed by 40 cycles of 95°C for 30 s (denaturation) and 60°C for 30 s (annealing) (Stedtfeld et al. 2018). Melting curve analysis was performed simultaneously using Wafergen software. Each qPCR run included no‐template controls, and all qPCR reactions were conducted in triplicate. Samples showing positive amplification in at least two technical replicates were considered successful and used for further analysis. HT‐qPCR experiments were performed by Hefei Yuanzai Biotechnology Co. Ltd. (Hefei, China).

### 2.7 Droplet Digital PCR Assay Setup

For comparisons between HT‐qPCR and ddPCR, a subset of 32 sampling sites was selected across six geographic regions (Table S7), and the same eDNA samples were subjected to parallel quantification by both methods. The 66 sample‐primer combinations included in the comparison were selected from HT‐qPCR positive detections, each representing one target species at one sampling site. The copy number concentrations (copies/L) quantified by each method were then compared.

Gene copy numbers were measured using the QX200 Droplet Digital PCR system (Bio‐Rad) with species‐specific primers for nine fish species. Each 20 μL reaction contained 10 μL of 2 × EvaGreen SuperMix, 1 μL of eDNA template, 0.25 μL of each primer, and 8.5 μL of nuclease‐free water. Droplets (~20,000) were generated using the QX200 AutoDG system. Each plate included two negative controls (nuclease‐free water) and one positive control (fish tissue DNA).

PCR Conditions: 95°C for 10 min, followed by 40 cycles of 94°C for 30 s, 60°C for 30 s, and 98°C for 10 min. Samples were analysed in triplicate. Post‐PCR, droplets were read using the QX200 droplet reader, and QuantaSoft (V1.7.4, Bio‐Rad) calculated concentrations (copies/μL) based on positive/negative droplet ratios, assuming a Poisson distribution. Results were adjusted for dilution to report eDNA concentration (copies/μL).

### 2.8 Data Analysis and Statistical Methods

Statistical analysis and visualization were performed using R software (version 4.4.0) (R Core Team 2024), including interactive visualizations were implemented using plotly (Sievert 2020) and ggplot2 (Wickham 2016).

#### 2.8.1 Efficiency and Sensitivity Analyses

For efficiency and sensitivity analyses of the HT‐qPCR detection system, boxplots were used to display the distribution of CT values across different plasmid DNA template concentration gradients, scatter plots to present data dispersion, and density plots to visually compare sensitivity differences, including the limit of detection (LoD) and limit of quantification (LoQ), among primers. Results are expressed as mean ± standard deviation (SD) calculated across replicates, with a significance level of *p* < 0.05.

#### 2.8.2 Regional Distribution and Species Detection Analyses

For comparisons across regions, eDNA distribution patterns of riverine fish were visualized using heatmaps (R package pheatmap) (Kolde 2019). Sankey diagrams were used to illustrate species detection across the watershed (R package networkD3) (Allaire et al. 2017). For each species‐region combination, occurrence was quantified as the detection rate, calculated as the number of sampling sites at which the species was detected divided by the number of effective sampling sites in that region. Effective sampling sites were defined as sites at which at least one fish species yielded a detectable eDNA HT‐qPCR signal; sites from which no fish species was detected were excluded from the denominator. A sampling site was considered positive for a given species if at least one amplification signal from any primer targeting that species was recorded. Detection rates were classified into three categories: High (0.6–1.0), Medium (0.3–0.6), and Low (0–0.3), represented as terminal nodes in the Sankey diagram.

To characterize the regional distribution of primer‐based eDNA detections, chord diagrams were constructed for each of the six study regions using the R package circlize (v0.4.x) (Gu et al. 2014). For each region, the raw HT‐qPCR detection records were merged with a primer–species reference table that assigned each of the 19 primers to its target fish species (2–3 primers per species). Records were then grouped by sampling site and fish species, and the number of primers yielding a positive eDNA signal within each site–species combination was calculated as the detection count. This count matrix (sampling sites × fish species) served as input for chordDiagram(). In the resulting diagrams, each ribbon connects a sampling site to a fish species; ribbon width is proportional to the number of species‐specific primers that tested positive at that site, and arc length reflects the cumulative primer detection count for each node across the region. Sampling sites were coloured using a pink‐to‐cyan gradient, and fish species were assigned fixed colours consistent across all six regional diagrams.

To further compare the quantitative eDNA signal across primers and species, a jitter scatter plot was generated using ggplot2. All six regional datasets were combined, and eDNA concentrations (copies/L) were log_10_‐transformed. Each data point represents one positive detection record, colour‐coded by fish species, with the x‐axis showing individual primer names and the y‐axis showing log_10_ (copies/L).

#### 2.8.3 Comparison Between HT‐qPCR and ddPCR

For comparisons between HT‐qPCR and ddPCR, the copy number concentrations (copies/L) quantified by each method were then compared. Deming regression (R package mcr) (Potapov et al. 2024) was applied to account for measurement errors in both methods, and Bland–Altman agreement analysis (R package BlandAltmanLeh) (Lehnert 2015) was used to assess systematic bias and interchangeability. Copy number data were log10‐transformed prior to analysis to meet the assumption of normality. Scatter plots with regression lines were used to visualize method comparison and correlation.

## 3 Results

### 3.1 Primer Design and Validation for Nine Freshwater Fish Species

A species‐specific primer database for 9 target fish species was established through literature review. Using searches combining “scientific name + quantitative PCR/digital PCR/primer” in PubMed and Web of Science, we identified 40 articles reporting primers for seven species including *C. carpio* and *H. nobilis* . After specificity evaluation, 2 high‐specificity primer pairs per species were selected. For *S. meridionalis* and *S. sinensis* , species‐specific primers were designed using mitochondrial genome sequences from GenBank (NC_014866.1, NC_056907.1). All primers were validated through qPCR efficiency testing (*R* ^2^ > 0.99) and product sequencing. Primer sequences, annealing temperatures, and sources are compiled in Table S5, forming the foundation for our HT‐qPCR detection system.

### 3.2 Development and Optimization of High‐Throughput qPCR Chip Assay

**FIGURE 2:**
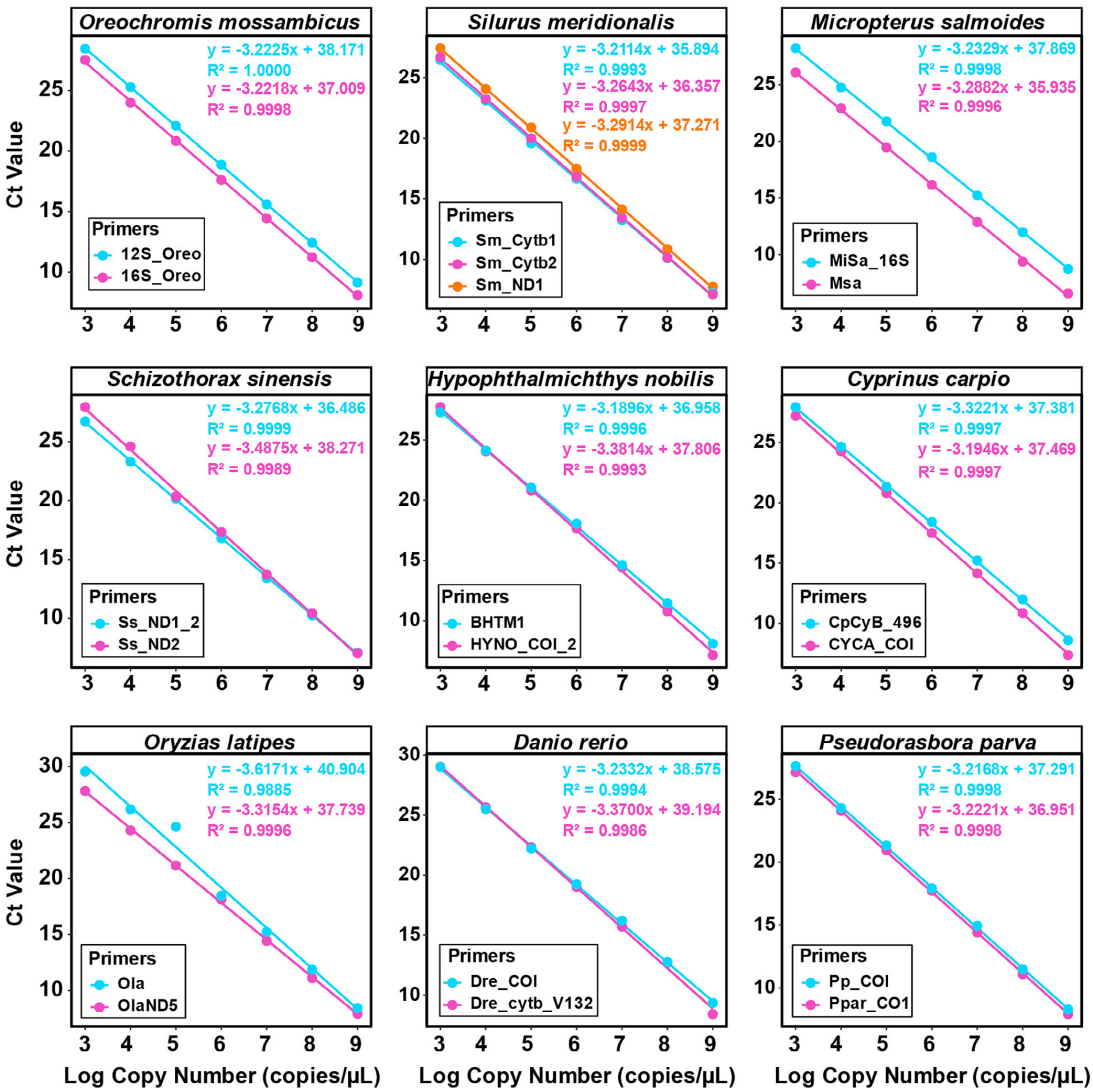
HT‐qPCR standard curves for nine fish species. Standard curves were generated using serial 10‐fold dilutions of species‐specific DNA templates spanning seven concentration levels (10^3^–10^9^ copies/μL). HT‐qPCR was performed at each dilution, and threshold cycle (Ct) values were recorded. The x‐axis represents log_10_ copy number (copies/μL) and the y‐axis represents Ct values. Each panel corresponds to one fish species; colours denote different primer pairs (see in‐panel legends). Data points represent measured Ct values; lines indicate linear regression fits.

Using serial dilutions of the plasmid standards constructed as described in Section 2.5 as templates, the HT‐qPCR detection system was employed to systematically evaluate 19 species‐specific primer pairs designed for 9 fish species, and standard curves were constructed. Experimental results demonstrated that all standard curves exhibited excellent linearity, with correlation coefficients (*R* ^2^) ranging from 0.9885 to 1.000 (Figure 2), confirming stable dose‐signal response characteristics in each reaction system. In stringent quality control experiments, no‐template controls (NTC) showed no specific amplification signals across all detection channels, robustly demonstrating the system's exceptional anti‐contamination capability and primer specificity.

### 3.3 Efficiency and Sensitivity of High‐Throughput qPCR Chip Assay

**FIGURE 3:**
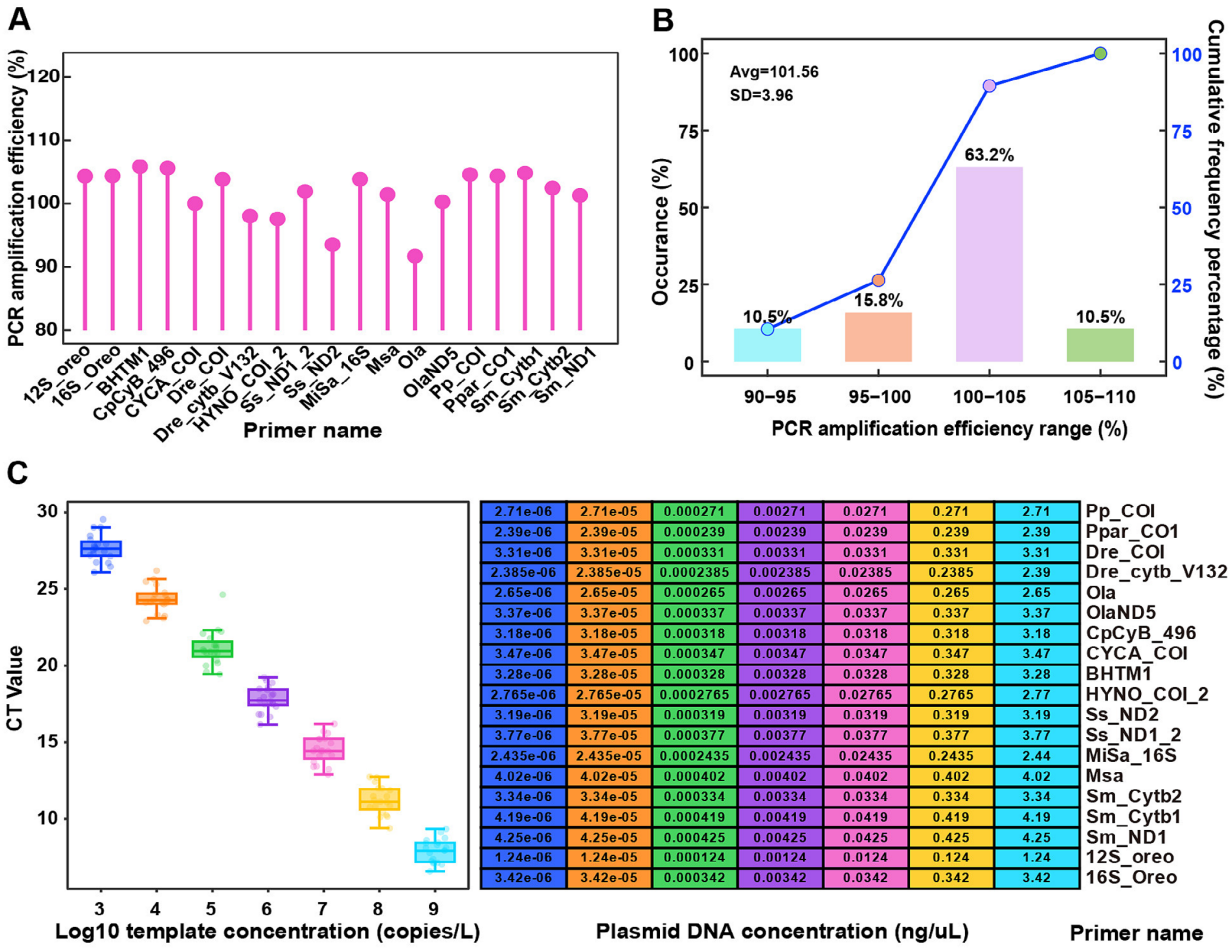
Development and validation of HT‐qPCR for fish species detection. (A) HT‐qPCR amplification efficiency of primers for 19 fish species. (B) Distribution of HT‐qPCR amplification efficiency of primers for 19 fish species. Bars represent the percentage of primers within each efficiency range (left y‐axis), and the line indicates the cumulative frequency (right y‐axis). (C) Sensitivity analysis of HT‐qPCR detection system. Boxplots with overlaid scatter points show the distribution of CT values for 19 fish species‐specific primers across serial dilutions of plasmid DNA standards (10^3^–10^9^ copies/L); corresponding plasmid DNA concentrations in ng/μL are shown in the right panel.

Based on the constructed amplification curve data, quantitative analysis of primer amplification efficiency demonstrated that all 19 primer pairs exhibited efficiencies within the optimal range of 91.07%–105.83% (Figure 3A), fully complying with internationally recognized qPCR primer efficiency standards (90%–110%). Specifically, amplification efficiencies displayed a gradient distribution pattern (Figure 3B): 2/19 of primers had efficiencies in the 90%–95% range, 3/19 in the 95%–100% range, 12/19 concentrated in the optimal 100%–105% range, and 2/19 reached the 105%–110% range. Notably, 17/19 of primers demonstrated efficiencies above 95%, with 15/19 concentrated in the optimal performance range of 95%–105%, comprehensively validating the excellent amplification performance of the primer system designed in this study.

To verify the sensitivity of the designed primers in HT‐qPCR, plasmid DNA gradient dilution experiments were conducted for 19 primer pairs (Figure 3C). As template concentration decreased, CT values of all primers increased systematically, with average CT values of approximately 7.91 at the highest concentration (10^9^ copies/μL) and 27.67 at the lowest concentration (10^3^ copies/μL). The CT values at each concentration gradient showed centralized distribution with small interquartile ranges, indicating good consistency in amplification efficiency among different primers. The scatter data point distribution in the box plots (Figure 3C) demonstrated good reproducibility across concentration gradients with minimal data fluctuation. At the 10^9^ copies/μL gradient, plasmid DNA concentrations ranged from 1.24 to 4.25 ng/μL. The LoQ values for all primers ranged between 10^2.62^–10^3.13^ copies/μL, and LoD values were significantly lower than quantification limits and showed a wider distribution range (10^0.34^–10^2.55^ copies/μL) (Figure S2).

### 3.4 Quantitative Assessment of Fish eDNA Across Six Regions in China

**FIGURE 4:**
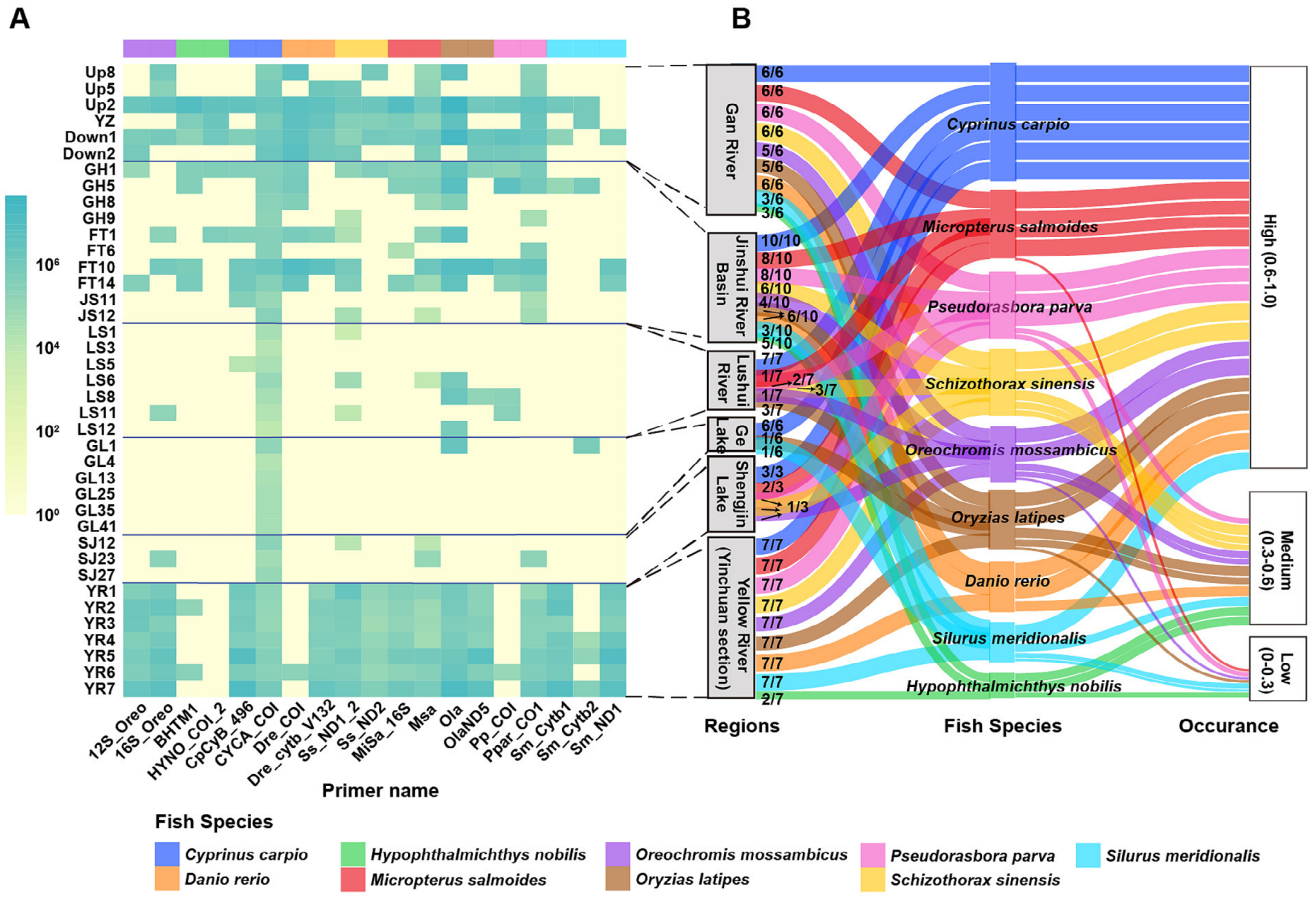
Regional variation in fish biodiversity assessed via eDNA HT‐qPCR. (A) Heatmap of absolute abundance of eDNA of fish in six regions. (B) Sankey diagram of eDNA detection rates of fish in six regions based on eDNA HT‐qPCR technology. For each species–region combination, a sampling site was considered positive if at least one amplification signal from any associated primer was recorded; detection rate was calculated as the ratio of positive sampling sites to effective sampling sites (see Methods for definition). Numbers annotated on the links indicate this ratio (e.g., 3/9). Detection rates were classified as High (0.6–1.0), Medium (0.3–0.6), or Low (0–0.3), shown as terminal nodes on the right.

We analyzed aquatic eDNA samples from 44 sites across 6 regions using HT‐qPCR technology and successfully quantified target fish eDNA in 39 sampling sites, systematically revealing significant differences in fish distribution among watersheds (Figure 4A). Samples from the GH and YR showed overall higher detection rates and eDNA concentration levels (light to dark blue), indicating rich fish diversity, while the LS, GH, and SJ generally exhibited lower fish eDNA concentrations (predominantly light colours), reflecting the relative scarcity of target fish species. Notably, the CYCA_COI primers for *C. carpio* were detected at all 39 sampling sites (100% detection rate), confirming the widespread distribution of this common economic fish species across various water systems in China. The detection results from Jinshui River were intermediate between high‐value and low‐value areas, displaying a more diverse species composition pattern. The high detection levels of *S. meridionalis* in certain specific watersheds reflected the adaptability and distribution preferences of specific species to geographical environments.

The results revealed significant differences in fish composition across different watersheds (Figure 4B). The GH watershed demonstrated the highest fish diversity and detection rates, with nearly all target fish species showing high detection rates, while GL and SJ exhibited relatively fewer detected fish species. *C. carpio* and *M. salmoides* showed high detection rates (> 0.6) across multiple watersheds, indicating their strong adaptability and wide geographical distribution. *S. sinensis* showed high detection rates in the GH, JS and YR. *O. mossambicus* was primarily detected at high rates in the GH and YR, demonstrating characteristic fish compositions in specific water bodies. Species with moderate detection rates (0.3–0.6) included *S. sinensis* and *P. parva* , which showed stable detection in specific watersheds, reflecting distinct regional distribution patterns. *O. latipes* , *D. rerio* , and *H. nobilis* showed low detection rates (< 0.3) in 2 lakes, possibly due to their low abundance in natural water bodies or limited distribution.

### 3.5 Analysis of Primer Differences in HT‐qPCR Results

**FIGURE 5:**
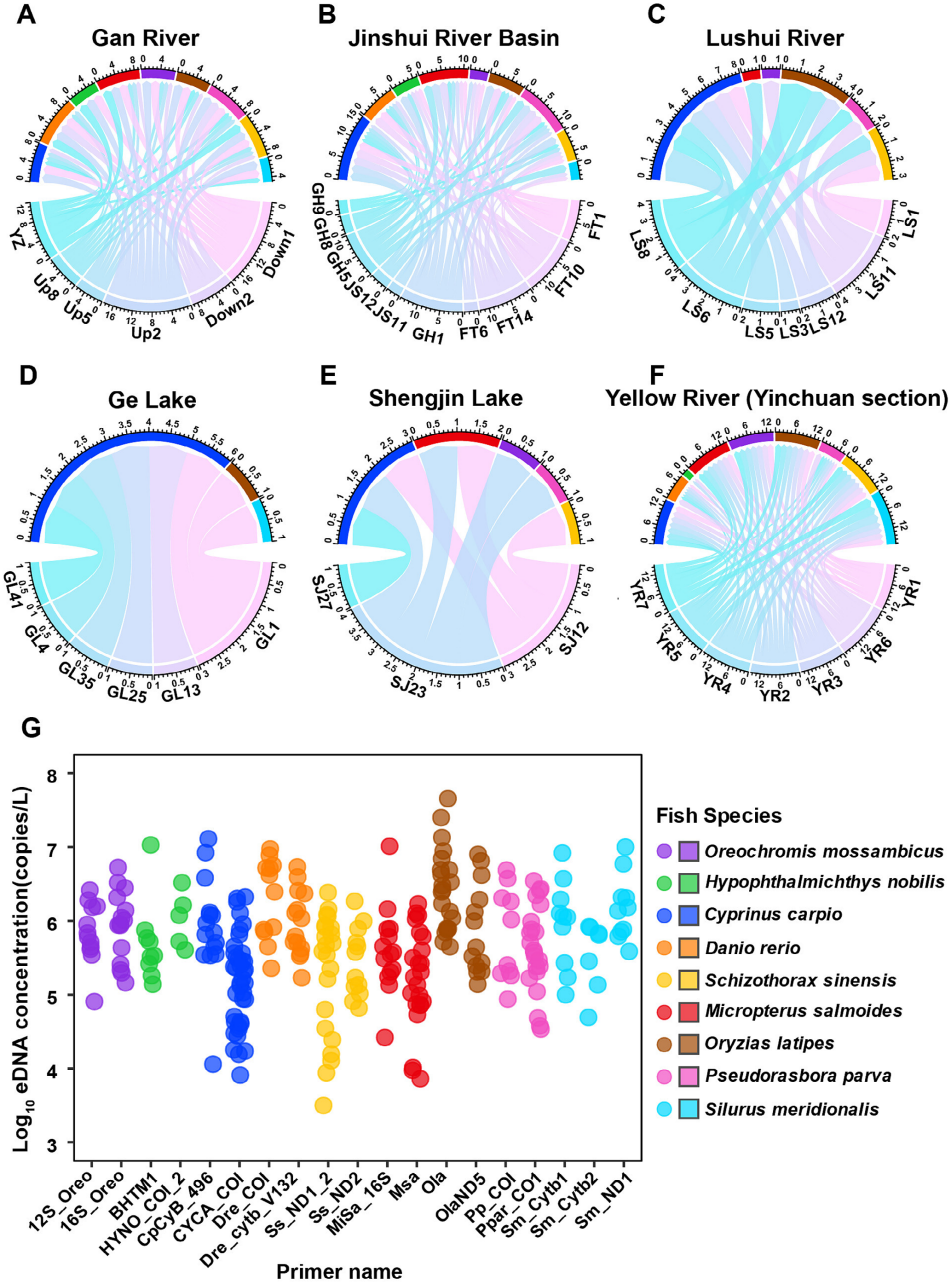
Multi‐primer eDNA detection patterns for nine fish species across six regions. (A–F) Chord diagrams illustrating the primer‐level detection relationships between sampling sites and fish species in six regions: (A) GH, (B) JS, (C) LS, (D) GL, (E) SJ, and (F) YR. Outer arc segments represent either sampling sites (pink‐to‐cyan gradient) or fish species (fixed colours consistent across all panels). Each ribbon connects a sampling site sector to a fish species sector; ribbon width is proportional to the number of species‐specific primers that yielded positive eDNA signals at that site, and arc length reflects the cumulative primer detection count for each node across the region. (G) Jitter scatter plot showing the operational detection range of all 19 primers across sampling sites in the six study regions. Each point represents one positive detection record from a single sampling site, colour‐coded by fish species. The x‐axis lists individual primer names grouped by target species, and the y‐axis shows log_10_‐transformed eDNA concentration (copies/L). The vertical spread of points within each primer reflects its detection range under real field conditions across sites and regions with varying fish abundance, while variation between co‐targeting primers for the same species indicates differences in amplification efficiency across mitochondrial loci.

Network connection diagrams visually represented the number and distribution of primers detected at each sampling site and further confirmed differences in fish distribution patterns across watersheds (Figure 5). The GH and JS exhibited the highest fish diversity with dense connection lines (Figure 5A,B); the LS and YR showed intermediate diversity (Figure 5C,F), while GL and SJ displayed sparse connection lines (Figure 5D,E). *C. carpio* , *S. sinensis* , *M. salmoides* , and *P. parva* were detected across multiple watersheds, demonstrating widespread distribution, whereas *O. mossambicus* , and *S. meridionalis* showed higher detection rates in specific watersheds, reflecting specialized habitat preferences.

Analysis of the operational detection range across different primers revealed eDNA concentrations spanning from 3.17E+03 to 4.53E+07 copies/L across all sampling sites and regions (Figure 5G). This wide range is expected, as the data were pooled from six geographically distinct regions encompassing sites with substantially different fish abundances, and therefore reflects the wild detection capability of each primer under diverse field conditions. Different primers targeting the same fish species generally exhibited similar concentration ranges, such as *O. mossambicus* (8.08E+04–5.29E+07 copies/L); however, some species like *C. carpio* showed considerable variation between primers (8.16E+03–1.29E+07 copies/L), reflecting differences in amplification efficiency and sensitivity among primers targeting different mitochondrial loci rather than methodological inconsistency, as all samples were processed using a standardized protocol under identical laboratory conditions. Certain primers for *O. latipes* fish demonstrated high detection sensitivity, reaching up to 4.53E+07 copies/L, while primers for *S. sinensis* generally yielded lower values, confirming the necessity of employing multi‐primer strategies in eDNA research to ensure comprehensive detection across the full range of eDNA concentrations encountered in natural water bodies.

Overall comparison across primers suggests that performance varied substantially across loci (Figure 5G). Among co‐targeting primers for the same species, those with higher detection frequencies and wider concentration ranges, such as Sm_Cytb1 for *S. meridionalis* , can be considered the better performing locus for routine monitoring applications. In contrast, primers for *S. sinensis* generally showed narrower detection ranges and lower median concentrations across sites, suggesting these loci may be less sensitive under the field conditions encountered in this study. These differences in locus level performance provide a basis for prioritizing primers in future survey designs when detection resources are limited.

### 3.6 Correlation Analysis Between High‐Throughput qPCR and ddPCR Quantification Results

**FIGURE 6:**
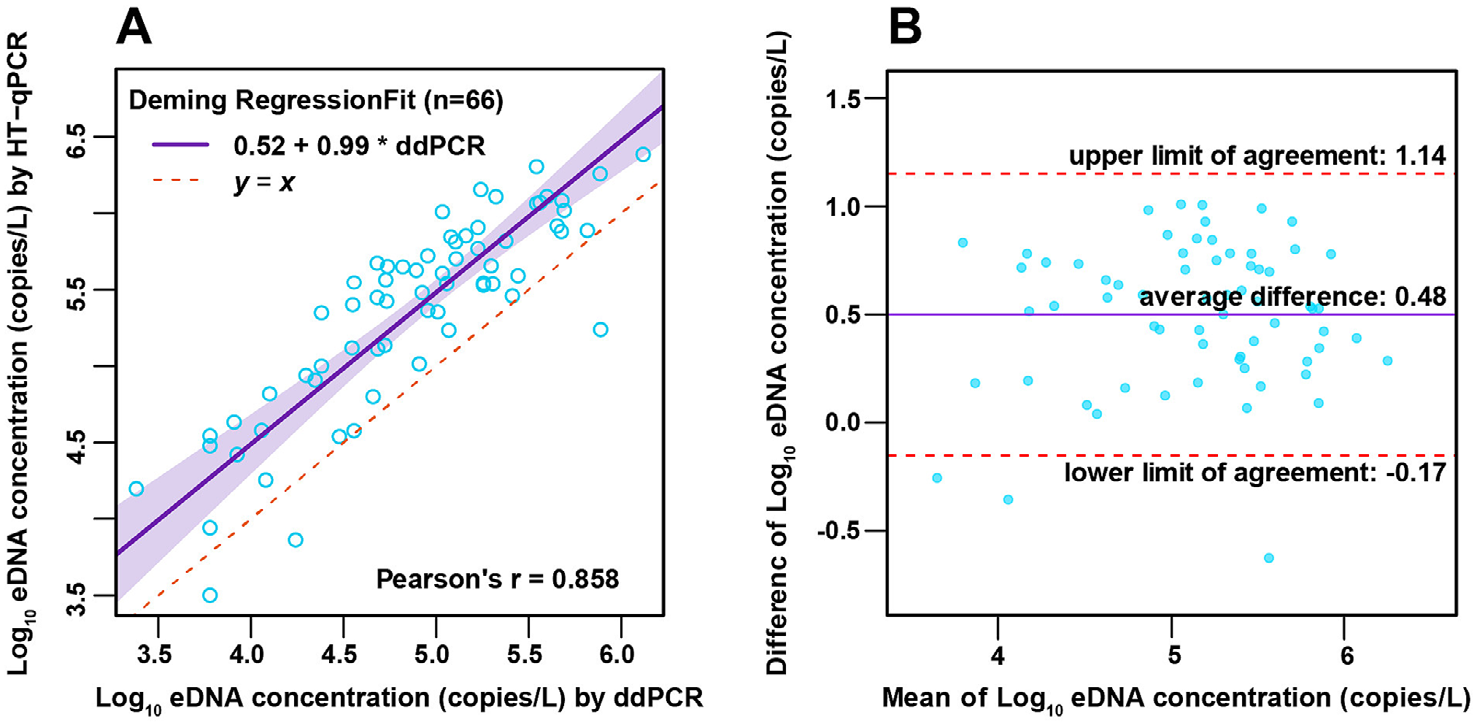
Quantitative comparison between HT‐qPCR and ddPCR for eDNA concentration measurements across six regions. Each data point represents a unique sample‐primer combination corresponding to one species detected at one sampling site (*n* = 66). (A) Deming regression analysis accounting for measurement error in both methods. The solid purple line represents the regression line, the shaded purple area indicates the 95% confidence interval, and the red dashed line denotes the identity line (y = x). (B) Bland–Altman analysis showing the relationship between the difference in measurements (HT‐qPCR minus ddPCR) and their mean. All data are presented on a log_10_‐transformed scale, with eDNA concentration expressed in copies/L.

To validate the quantitative reliability of HT‐qPCR, a subset of 32 sampling sites was selected for parallel ddPCR analysis, yielding 66 sample‐primer combinations in total. The quantification results from both methods were compared (Figure 6). All 66 combinations yielded positive ddPCR results, confirming quantitative concordance between the two methods within the validated subset.

Deming regression analysis (Figure 6A) revealed a good linear relationship between the two methods (Pearson *R* ^2^ = 0.858), with a regression equation of HT‐qPCR = 0.52 + 0.99 × ddPCR, indicating highly correlated measurements between the two methods. The regression line (purple) was slightly higher than the identity line (red dashed line), suggesting that HT‐qPCR measurements were generally higher than ddPCR measurements.

Bland–Altman agreement analysis (Figure 6B) more intuitively demonstrated the systematic difference between the two methods. The mean difference was 0.48 log units (purple line), indicating that HT‐qPCR measurements were on average approximately 10^0.48^≈3 times higher than ddPCR measurements. The 95% limits of agreement ranged from −0.17 to 1.14, with most data points evenly distributed around the mean difference line, showing no obvious concentration‐dependent trends. This indicates that this systematic difference remained relatively stable across different concentration levels.

It should be noted that ddPCR achieves absolute quantification via Poisson statistics without relying on a standard curve, whereas HT‐qPCR depends on a plasmid‐based standard curve that may not fully represent the complexity of real environmental samples. The comparison presented here therefore reflects quantitative method agreement rather than detection sensitivity. Despite the systematic offset, the two methods showed strong correlation and consistent bias, allowing for data conversion through a simple correction factor. These results support the use of HT‐qPCR as a reliable high‐throughput quantification approach for large‐scale aquatic biodiversity monitoring.

## 4 Discussion

### 4.1 Effectiveness of Multi‐Primer Approach for Species‐Specific Detection

At the catchment scale, when quantifying fish populations using eDNA coupled with HT‐qPCR, estimates for the same species vary with the mitochondrial locus targeted (e.g., 12S, COI, Cytb) because primer amplification efficiency and specificity differ across loci (Figure S3). For most species (e.g., *O. mossambicus* , *H. nobilis* , *C. carpio* , *D. rerio* ), co‐targeting primers generally detected the same sampling sites, indicating that the two approaches captured consistent spatial patterns of species occurrence even when absolute eDNA concentrations differed between primers. The bar heights at each sampling site are very similar, indicating that these primers have comparable amplification efficiencies and detection capabilities. The wide eDNA concentration range observed in Figure 5G, spanning more than four orders of magnitude, represents the operational detection range of each primer under real field conditions across six regions with substantially different fish abundances rather than methodological inconsistency, as all samples were collected and processed under a standardized protocol. The variation in detection range between co‐targeting primers for the same species, most evident in *C. carpio* , reflects differences in amplification efficiency across mitochondrial loci. This primer‐specific performance heterogeneity further reinforces the value of multi‐primer designs, as combining primers with complementary detection ranges achieves broader and more reliable coverage than any single primer can provide.

Analytical sensitivity, quantified as LoD and LoQ for each primer (Figure S2), offers a laboratory basis for understanding why certain primers outperform others under field conditions (Klymus et al. 2020). LoD spanned nearly three orders of magnitude across the 19 primers (2.19 to 358.32 copies/μL), whereas LoQ was far more constrained (420 to 1349 copies/μL, roughly a threefold range). This asymmetry suggests that most primers reach comparable quantification precision once template concentration is sufficient, but they diverge sharply in how little eDNA they can reliably detect. The observed LoD variation reflected the specificity of individual primer pairs against environmental background eDNA rather than any systematic property of the mitochondrial locus targeted (Thalinger et al. 2021). The within species paired comparisons illustrate this clearly. For *O. latipes* , OlaND5 and Ola target amplicons of identical length (108 bp) yet differ fivefold in both LoD and LoQ, attributing the performance gap to locus rather than fragment size. For *D. rerio* , the shorter Dre_COI (73 bp) performed worse than the longer Dre_cytb_V132 (132 bp) in both metrics, showing that shorter amplicons do not guarantee better sensitivity when primer specificity differs (Stadhouders et al. 2010). The *P. parva* pair is the most striking case: two COI primers differ in LoD by a factor of 33 (4.88 vs. 164.62 copies/μL) yet their LoQ values are nearly identical (875 vs. 892 copies/μL), indicating that the weaker primer generates spurious signal specifically at trace concentrations while retaining normal quantitative accuracy at higher loads (Lefever et al. 2013).

Except for *Silurus meridionalis* , there is a distinct trend where only Sm_Cytb1 shows stable and high detection levels across sites. The other primers (e.g., Sm_Cytb2 or Sm_ND1) likely show significantly lower concentrations, unstable detection, or complete absence at some sites. This indicates a significant performance discrepancy between the designed primers for this species. *Silurus meridionalis* populations may have genetic variations (haplotypes) that differ from the reference sequence used to design the primers. If Sm_Cytb2 or Sm_ND1 primers land on a site with a mutation in the local population, amplification efficiency can drop drastically or fail completely. Studies have shown that even a single mismatch at the 3′ end of a primer can inhibit PCR. This is particularly common in species with high genetic diversity or when primers are designed based on databases that may not reflect the local genotype (Collins et al. 2019). To minimize such lineage‐specific amplification biases, a good practice in primer design is to incorporate representative sequences from multiple evolutionary lineages or haplotypes during primer development. eDNA in water is often degraded into short fragments. Sm_Cytb1 targets a shorter amplicon (140 bp) and Sm_Cytb2 targets a longer one (213 bp); the shorter target will be more reliably detected because it persists longer in the environment. A previous study demonstrated that primers targeting shorter fragments yield significantly higher detection rates in eDNA surveys (Deagle et al. 2006). Conversely, longer fragments, though less stable, show a stronger correlation with hydroacoustic data, suggesting they more accurately reflect living fish biomass (Jo et al. 2017). Therefore, leveraging these length‐dependent differences offers significant potential for improving the accuracy of species biomass estimation.

The Ss_ND1_2 and Ss_ND2 primers both bind conserved regions of the mitochondrial ND genes and, from alignment with six congeneric Schizothorax sequences, are unlikely to separate *S. sinensis* from close relatives. We therefore treat them as Schizothorax‐level markers assigned to *S. sinensis* , not as species diagnostic assays. In our previous study, metabarcoding of the same eDNA material (Cheng et al. 2024) recovered *S. sinensis* at most sites of JS, with few reads from other Schizothorax spp. In this study, Ss_ND1_2 amplified at six of ten JS sites (Figure 4), broadly in line with that result; Ss_ND2 was positive at only one site (GH1), which we attribute mainly to lower assay performance in environmental DNA rather than a different fish assemblage. This does not remove the in silico specificity concern, but in JS where *S. sinensis* appears to dominate the schizothoracin eDNA pool, the two datasets together make it reasonable to link Ss_ND1_2 positives to this species. Where several Schizothorax congeners coexist, amplicon sequencing or SNP‐targeted probes would still be needed for firm species‐level assignment.

### 4.2 Performance Comparison Between HT‐qPCR and ddPCR

This study systematically compared the performance differences between HT‐qPCR chip technology and ddPCR in eDNA quantification through Deming regression analysis and Bland–Altman agreement analysis. The results demonstrated good linear correlation between the two methods (*r* = 0.858) but revealed systematic bias with HT‐qPCR measurements averaging approximately 3‐fold higher than ddPCR. This systematic bias primarily stems from fundamental differences between the two technologies: ddPCR achieves absolute quantification through sample partitioning and Poisson statistics without relying on standard curves (Guri et al. 2024), while HT‐qPCR still requires indirect quantification based on standard curves (Waseem et al. 2019). A comparison of qPCR and ddPCR across three teleost fish eDNA assays found that both methods yield positively correlated concentration estimates across a range of DNA concentrations, supporting the strong linear correlation observed in the present study (Guri et al. 2024). Furthermore, the plasmid‐based standard curve used in HT‐qPCR is constructed under idealized conditions and may not adequately account for PCR inhibitors, matrix effects, or DNA degradation commonly present in real environmental water samples, which could contribute to the observed overestimation relative to ddPCR. ddPCR exhibits superior sensitivity and quantification precision at low DNA concentrations (< 1 copy/μL) due to its partitioned reaction mechanism that effectively reduces the impact of PCR inhibitors and provides more stable quantification results (Huggett et al. 2013).

Regarding precision and linear dynamic range, both technologies exhibit distinct advantages. ddPCR demonstrates superior precision and sensitivity for detecting small fold changes in gene expression, particularly excelling in applications involving low abundant targets with expression differences of 2‐fold or lower, where conventional qPCR often produces highly variable and non‐reproducible results (Taylor et al. 2017). Particularly for low‐concentration eDNA samples, ddPCR shows significant superiority in quantification precision (Doi, Uchii, et al. 2015), which is crucial for monitoring rare and invasive species (Doi, Takahara, et al. 2015). However, HT‐qPCR technology exhibits clear advantages in dynamic range, with the linear range typically spanning 5–6 orders of magnitude when properly validated (Waseem et al. 2019). HT‐qPCR technology significantly enhances detection throughput through nanoliter‐scale reaction volumes and automated mixing systems, capable of simultaneously processing up to 96 assays and 96 samples in a single chip (Waseem et al. 2019).

From a practical application perspective, technology selection should be based on specific research objectives and sample characteristics. For studies requiring high‐precision detection of rare species or low‐concentration eDNA, the absolute quantification capability of ddPCR without standard curves and superior resistance to inhibitors makes it the preferred method, particularly demonstrating higher accuracy in fish population abundance estimation (Doi, Uchii, et al. 2015). The systematic bias observed in this study exhibits good predictability and stability, allowing data conversion through established correction factors, which provides possibilities for complementary application of both technologies. However, the 95% limits of agreement (−0.17 to 1.14 log units) reveal substantial variability at the individual level, with the ratio between the two methods ranging from approximately 0.68 to 13.8‐fold across individual sample‐primer combinations. This range indicates that the mean correction factor of approximately 3‐fold is a population‐level estimate rather than a constant applicable to all primer and species combinations. As discussed in Section 4.1, amplification efficiency varies considerably among primers, even when targeting the same mitochondrial locus within the same species. Such primer‐specific performance differences are likely a major source of the variability observed in the method bias. Caution is therefore warranted when applying a uniform correction factor across different species or primer sets, and primer‐specific calibration is recommended for studies requiring high quantitative precision. This assumption reflects the theoretical advantage of ddPCR's quantification through partitioning, rather than direct validation against an independent ground truth such as independently quantified plasmid standards. A true comparison against a known reference would be needed to fully resolve the quantitative bias between the two methods. Similar concordance between HT‐qPCR and ddPCR has also been reported in other environmental water monitoring contexts. In a study comparing conventional qPCR, ddPCR, and HT‐qPCR for antibiotic resistance gene profiling in tropical water systems, conventional qPCR and ddPCR yielded highly concordant quantification results, while HT‐qPCR was most effective for broad multi‐target screening and ddPCR was superior for detecting rare or low‐abundance targets (Srathongneam et al. 2026). These methodological findings parallel the performance patterns observed in the present study. Future research should further explore how to combine the advantages of both technologies to establish a more comprehensive eDNA quantification detection system. Users should nonetheless remain aware of these inherent limitations when interpreting absolute quantification values derived from field eDNA samples.

### 4.3 Implications for Freshwater Biodiversity Monitoring and Conservation

HT‐qPCR provides an efficient technological bridge between catchment‐scale eDNA sampling frameworks and the decision‐grade quantitative information required for conservation and fisheries management. eDNA, combined with molecular methods, is now widely applied to monitor freshwater biodiversity in rivers and to characterize its spatiotemporal patterns. In this context, HT‐qPCR platforms operate in nanoliter volumes and use combinatorial matrices to simultaneously process multiple assays across many samples, markedly increasing analytical throughput, conserving reagents, and automating workflows while maintaining quantitative accuracy. In this study, the Wafergen SmartChip was configured in an 80‐sample × 64‐assay format, generating 5120 reactions in a single run. The equivalent workload using conventional 96‐well qPCR would require approximately 57 separate plate runs, as each of the 19 assays run in triplicate across 80 samples exceeds the capacity of a single plate. ddPCR also uses a 96‐well format and can accommodate all 80 samples in a single run, but processes only one assay per run, requiring 19 separate runs to cover all 19 primer pairs. Beyond run efficiency, each sample consumes only approximately 6.4 μL of input on the HT‐qPCR chip, compared with approximately 1140 μL for the equivalent conventional qPCR workflow. This reduction in sample consumption is particularly valuable in eDNA studies where extracted volume is limited. ddPCR carries the highest per‐reaction consumable cost owing to its proprietary droplet generation reagents and cartridges. These combined advantages in throughput, sample economy, and cost make large, repeated monitoring campaigns economically feasible, and allow HT‐qPCR to serve as a targeted complement to ddPCR when predefined indicator species or management targets must be tracked consistently across space and time.

Methodological innovation is central to realizing these benefits. HT‐qPCR has been validated in water‐quality and environmental applications for parallel quantification of multiple targets across many samples. In freshwater biodiversity research, this architecture can be repurposed to build species‐specific panels for priority taxa. For large‐scale biodiversity monitoring projects, HT‐qPCR chip technology offers significant advantages in cost‐effectiveness, detection throughput, and operational convenience, enabling rapid and economically efficient shellfish species simultaneous detection (van der Pouw Kraan et al. 2024). In fisheries management, basin‐wide assessments increasingly rely on eDNA to depict diversity and abundance gradients over hundreds of kilometres; results are consistent with traditional surveys while covering much larger spatial extents (Elmore et al. 2024).

A broader question is whether HT‐qPCR could replace metabarcoding in freshwater biodiversity monitoring. The two methods are best viewed as complementary tools with distinct strengths. Metabarcoding is untargeted and capable of revealing unexpected or previously unrecorded taxa, making it well suited for community level biodiversity assessment (Liu et al. 2025). HT‐qPCR is limited to predefined targets but offers species‐specific absolute quantification with high sensitivity, which is particularly valuable for tracking indicator species or management‐relevant taxa across space and time. Neither method replaces the other; the choice depends on whether the monitoring objective is broad community characterization or targeted quantitative surveillance of a defined species panel.

HT‐qPCR delivers rapid, reproducible, and cost‐effective quantification to support adaptive management, including rapid response to invasions, compliance monitoring for fishing bans, and evaluation of targeted restoration outcomes. Overall, integrating catchment‐scale sampling logistics with robust, policy‐oriented quantification strengthens freshwater conservation and governance.

### 4.4 Future Applications and Research Directions of FishChip

FishChip holds considerable potential for integration into large‐scale freshwater biomonitoring programs. The simultaneous quantification of multiple species in a single run makes it well suited for regulatory frameworks such as the EU Water Framework Directive, where most fish assessment metrics are expressed as richness or relative abundance and are therefore compatible with eDNA‐derived data (Pont et al. 2021). Beyond routine monitoring, FishChip could also be used for the early detection of invasive species across river networks and for tracking seasonal changes in fish community composition. The individual species‐specific assays can also be deployed on conventional qPCR instruments where HT‐qPCR platforms are not available, broadening accessibility (van der Pouw Kraan et al. 2024). Nevertheless, several challenges remain. Expanding the species panel requires the design and rigorous validation of species‐specific primers for each additional target, which is both time‐ and resource‐intensive. Incomplete or error‐prone reference databases carry a risk of false positives (van der Pouw Kraan et al. 2024), and translating eDNA copy numbers into reliable abundance estimates requires species‐specific calibration under varying environmental conditions. Standardization of sampling protocols and analytical workflows across laboratories and geographic regions will be essential before FishChip can be routinely implemented in national monitoring programmes.

## 5 Conclusion

Collectively, the experimental data demonstrate that the established HT‐qPCR environmental DNA (eDNA) detection system meets international benchmarks for methodological reliability, primer specificity, and amplification efficiency, delivering an efficient, non‐invasive quantitative pathway for aquatic ecosystem monitoring. Quantification is highly concordant with droplet digital PCR (ddPCR) results (*r* = 0.858); although HT‐qPCR exhibits a stable ~3‐fold positive bias in absolute copy number relative to ddPCR, this can be corrected with a unified calibration factor to enable cross‐platform data integration. Detection‐rate heterogeneity among primers targeting the same species demonstrates the necessity of multi‐primer panels to enhance robustness, and differences in fish assemblages among sampling sites within the same watershed reflect microenvironmental effects. Overall, this system provides comparable and reliable evidence for fish‐resource conservation and management and, by virtue of its high throughput, low cost, and scalability, holds strong promise for routine basin‐wide monitoring and rapid early warning.

## Author Contributions

**Shoutao Cheng:** data curation, formal analysis, visualization, writing – original draft, writing – review and editing. **Jiasheng Zhang:** formal analysis. **Zhuming Liang:** formal analysis. **Hao Xue:** formal analysis. **Lingsong Zhang:** resources, funding acquisition. **Yeyao Wang:** supervision, methodology, writing – review and editing. **Fansheng Meng:** supervision, funding acquisition, writing – review and editing. All authors have read and agreed to the published version of the manuscript.

## Funding

This work was supported by the National Key Research and Development Program of China (2021YFC3200100) and the Joint Research Project II on Ecological Environment Protection and Restoration of the Yangtze River (2022‐LHYJ‐02‐0506‐09).

## Ethics Statement

This study did not involve experimentation on live animals. Fish tissue used for constructing the plasmid standards was obtained from fish that died of natural causes during routine aquaculture and were not captured, handled, or sacrificed for the purposes of this study. Environmental DNA (eDNA) samples were collected from water bodies through non‐invasive sampling and did not involve the capture or handling of live fish. Accordingly, institutional animal care and use approval was not required for this study.

## Conflicts of Interest

The authors declare no conflicts of interest.

## Supporting information


**Table S1:** Information on sampling sites for water samples in the basin.
**Table S2:** Information on sampling sites for water samples in the basin.
**Table S3:** Mitochondrial genome information of nine fish.
**Table S4:** Congeneric species considered during species‐specific primer design for mitochondrial genome sequence alignment.
**Table S5:** Fish primer sequence information.
**Table S6:** BLASTN alignment results of cloned plasmid inserts against the NCBI GenBank database.
**Table S7:** Information of 32 sampling sites for ddPCR experiments.
**Figure S1:** Representative field photographs of the sampling sites across the studied area. (A) Gan River. (B) Jinshui River Basin. (C) Lushui River. (D) Ge Lake. (E) Shengjin Lake. (F) Yinchuan section of the Yellow River.
**Figure S2:** Limit of Detection (LOD) and Limit of Quantification (LOQ) of HT‐qPCR method.
**Figure S3:** Performance Evaluation of Different eDNA Primers for Single Fish Species at Various Locations.

## Data Availability

The original contributions presented in this study are included in the article/Supporting Information—S1. Primer sequences used in this study are provided in the Supporting Information—S1. High‐throughput quantitative PCR (HT‐qPCR) and droplet digital PCR (ddPCR) data, including copy number results used for analysis, are stored on the Dryad repository (DOI: 10.5061/dryad.fqz612k6v). Further inquiries can be directed to the corresponding author.
