# Supplementary material for: FishChip: Development of a High‐Throughput qPCR Assay for Freshwater Fish eDNA Quantification and Its Comparison With Droplet Digital PCR
Source: Mol Ecol Resour. 2026 Aug 5;26(6):e70187. doi: 10.1111/1755-0998.70187 (PMC13441544; doi:10.1111/1755-0998.70187)
Supplement: Supplementary file 1 [file MEN-26-e70187-s001.docx]

**Supplementary material**

**Text S1 - S8**

**Text S1** **Sampling Sites**

These sampling areas included: the Jinshui River Basin (10 sampling sites) and Lushui River (7 sampling sites) in Hubei Province, both significant tributaries of the Yangtze River; the Gan River (6 sampling sites) within the Jinshui River watershed; the Yinchuan section of the Yellow River in Yinchuan City, Ningxia (7 sampling sites), a representative segment of the middle reaches of the Yellow River; Lake Gehu in Jiangsu Province (9 sampling sites), a key lake within the Taihu Lake basin; and Lake Shengjin in Chizhou City, Anhui Province (5 sampling sites), a critical wetland lake in the middle and lower reaches of the Yangtze River that is also designated as a national nature reserve.

**Text S2 Taxonomic classification of the purchased fish**

To verify the taxonomic classification of the purchased live fish, genomic DNA was first extracted from fish tissues purchased online and used as a template for PCR amplification with the universal primer MiFish-U (F:5'-GTCGGTAAAACTCGTGCCAGC-3', and R:5'-CATAGTGGGGTATCTAATCCCAGTTTG-3'). The amplified products were gel-purified and subjected to Sanger sequencing. The sequencing results were then compared through bioinformatics analysis to ultimately determine the mitochondrial genome information of the obtained fish species (see Table S3 for details).

**Text S3 Primer selected and design**

To obtain species-specific primer information for fish, a systematic literature search was first conducted, and the retrieved primers were screened. For fish species without available primers in the literature, primers were independently designed based on mitochondrial genomic information of the target fish.

**1. Experimental Primer Screening**

To acquire species-specific primer information for target fish, a combined approach of systematic literature retrieval and bioinformatics analysis was employed:

(1) Searches were performed on PubMed and Web of Science databases using species Latin names combined with keywords such as "qPCR primers" and "ddPCR assay." An example search query was: ("*Cyprinus carpio*"[Title/Abstract]) AND ("quantitative PCR" OR "ddPCR").
(2) Priority was given to validated primers published in JCR Q1 and Q2 journals within the last five years.
(3) The frequency of primer occurrence in the retrieved literature was calculated, and the two most frequently cited primer pairs were selected for use in this study.

**2. Experimental Primer Design**

For fish species not identified in the literature search, species-specific primers were independently designed based on mitochondrial genomic information. To ensure primer specificity, mitochondrial genome sequences of the target fish species and congeneric species available in the NCBI database were downloaded, and multiple sequence alignment was performed to identify interspecific variable regions and avoid highly conserved regions that might result in non-specific amplification. For species rich genera such as *Schizothorax*, where including all congeneric species was impractical, the most closely related species to the target species were selected for sequence alignment based on a published mitochondrial phylogenetic framework (Qin et al., 2024) These species pose the greatest risk of cross-amplification and thus represent the most stringent test of primer specificity. The congeneric species considered for each target fish are summarized in Table S4. Design focused on mitochondrial regions including Cytb, COI, ND, 12S, and 16S. Primer design was primarily conducted using Primer Premier 6 software (Premier, Canada) (Ye et al., 2012).

To verify primer specificity, designed primers were validated using the Primer-BLAST tool on the NCBI website against the NCBI nucleotide collection (nr) database, with the target organism set to bony fish (taxid:7898). Primers were synthesized by Tsingke Biotechnology Co., Ltd. (Beijing, China), with PAGE purification method selected. Primer design adhered to the following parameter standards (*Design of Primers and Probes for Quantitative Real-Time PCR Methods*, n.d.):

(1) Primer length ranged from 18 to 24 bases.
(2) Expected annealing temperature (Tm) was (58±2) °C.
(3) GC content was between 40% and 60%.
(4) Amplification product length ranged from 70 to 250 bases.
(5) Formation of primer dimers and hairpin structures was avoided.
(6) The 3' end of the primer was in regions with high interspecies variation, with at least 2-3 base-specific differences.

**Text S4 Standard Material Conduction**

To ensure the quantitative accuracy of high-throughput quantitative PCR (HT-qPCR) technology, this study first obtained plasmid standards through TA cloning. Plasmid standards were constructed in-house via TA cloning. Briefly, target DNA fragments were amplified from genomic DNA of each target fish species using species-specific primers (Table S5). PCR products were verified by agarose gel electrophoresis, ligated into the pMD™19-T vector (TaKaRa, Japan), and transformed into E. coli DH5α competent cells. Positive clones were confirmed by Sanger sequencing (Tsingke Biotechnology, Beijing, China), and sequence identity was verified by BLASTN alignment against the NCBI GenBank database (identity≥98%; results summarized in Table S5). Purified plasmid DNA was quantified by spectrophotometry and serially diluted to generate standard curves for HT-qPCR quantification.

**Text S5 Plasmid Copy Number Calculation Method**

This experiment employed molecular quantification to calculate plasmid copy numbers, using the following Equation 1:

$Copy number（copies/\mu l） = \frac{C \times N_{A}}{M_{W}\times{10}^{9}}$	Equation 1

Where:

1. *C* is the plasmid DNA concentration (unit: ng/μL).
2. NA=6.022×10^23^ mol^−1^ (Avogadro's constant).
3. *Mw*=*L_total_*×660 g/(mol\cdotpbp) (molecular weight of the plasmid).
4. *L_total_*=*L_vector_*+*L_insert_* (total length of the plasmid, unit: bp).

**Text S6 Amplification Efficiency Validation**

The association between Ct values and the logarithm of template copy numbers was analyzed using linear regression in R. Amplification efficiency was calculated as shown in Equation 2. Validity was determined by meeting the following criteria simultaneously: amplification efficiency of 80%-120%, standard curve R^2^ ≥ 0.99, Ct values decreasing progressively across the dynamic range, and no amplification in negative controls (Ct ≥ 35).

$Effeciency={10}^{（1/slope）}-1$	Equation 2

**Text S7 Amplification Specificity Validation**

Plasmid DNA from nine target fish species was selected, and specificity validation was performed using the same primer set for each species, with three technical replicates (n=3) per species. Negative controls (no-template controls) and positive controls (plasmid DNA standards) were also included.

After amplification, a melting program from 65°C to 95°C (ramp rate: 0.1°C/s) was conducted, and fluorescence signals were collected using the Wafergen SmartChip system to analyze the melting curve characteristics. Data analysis criteria were as follows:

1. The melting curve should exhibit a single characteristic peak with a peak width ≤1.5°C.
2. The Ct value for the target species should be ≤33, with amplification efficiency between 80-120%.
3. The Ct value for non-target species should be ≥35 or undetected.
4. No amplification signal should be observed in the negative control (no-template control).

**Text S8 Amplification Sensitivity Validation**

Sensitivity was assessed by determining the limit of detection (LoD) and limit of quantification (LoQ). LoD is defined as the lowest amount of target gene that can be reliably detected (Y. Zhao et al., 2019), while LoQ is the lowest amount of target gene that can be quantified using the standard curve (Zhu et al., 2022). According to the Clinical and Laboratory Standards Institute (CLSI) EP17-A2 guidelines (Fuentes-Arderiu, 2002), LoD and LoQ were determined based on positive amplification in at least five out of six replicates.

Specifically, LoD was identified by analyzing a series of analyte concentrations until the lowest concentration that could be significantly distinguished from zero with 95% confidence was found. LoQ was defined as the lowest quantifiable concentration with acceptable performance, requiring a relative standard deviation (RSD) of less than 20% in replicate experiments.

**Table S1 - S5**

**Table S1** Information on sampling sites for water samples in the basin

| Date | Basin | Aquatic Ecosystem Types | Sample Name | Longitude | Latitude |
| --- | --- | --- | --- | --- | --- |
| 2022.08 | Gan River | River | Up8 | 29.90434 N | 114.25873 E |
|  |  |  | Up5 | 29.92387 N | 114.24882 E |
|  |  |  | Up2 | 29.93070 N | 114.24337 E |
|  |  |  | YZ | 29.93536 N | 114.21041 E |
|  |  |  | Down1 | 29.94167 N | 114.19858 E |
|  |  |  | Down2 | 29.95032 N | 114.19824 E |
| 2023.07 | Jinshui River Basin | River | GH1 | 29.71858 N | 114.38535 E |
|  |  |  | GH5 | 29.80516 N | 114.34657 E |
|  |  |  | GH8 | 29.91788 N | 114.30845 E |
|  |  |  | GH9 | 29.93606 N | 114.20740 E |
|  |  | Lake | FT1 | 29.96685 N | 114.21022 E |
|  |  |  | FT6 | 29.99578 N | 114.19890 E |
|  |  |  | FT10 | 30.02094 N | 114.21174 E |
|  |  |  | FT14 | 30.07219 N | 114.19808 E |
|  |  | River | JS11 | 30.13462 N | 114.14880 E |
|  |  |  | JS12 | 30.31348 N | 114.13041 E |
| 2023.07 | Lushui River | River | LS1 | 29.89646 N | 113.66506 E |
|  |  |  | LS3 | 29.70894 N | 113.88930 E |
|  |  |  | LS5 | 29.53477 N | 114.03942 E |
|  |  |  | LS6 | 29.45757 N | 113.95047 E |
|  |  |  | LS8 | 29.38692 N | 113.87902 E |
|  |  |  | LS11 | 29.16402 N | 113.71979 E |
|  |  |  | LS12 | 29.20961 N | 113.91344 E |
| 2023.08 | Ge Lake | Lake | GL1 | 31.50595 N | 119.76190 E |
|  |  |  | GL2 | 31.51991 N | 119.77168 E |
|  |  |  | GL4 | 31.55169 N | 119.77528 E |
|  |  |  | GL5 | 31.55090 N | 119.79518E |
|  |  |  | GL13 | 31.58068 N | 119.77250 E |
|  |  |  | GL25 | 31.61765 N | 119.85729 E |
|  |  |  | GL32 | 31.68669 N | 119.81826 E |
|  |  |  | GL35 | 31.65455 N | 119.86976 E |
|  |  |  | GL41 | 31.65396 N | 119.81071 E |
| 2023.11 | Shengjin Lake | Lake | SJ12 | 30.39437 N | 117.05628 E |
|  |  |  | SJ16 | 30.36346 N | 117.10635 E |
|  |  |  | SJ17 | 30.38968 N | 117.09914 E |
|  |  |  | SJ23 | 30.40152 N | 117.12249 E |
|  |  |  | SJ27 | 30.43197 N | 117.15697 E |
| 2024.07 | Yinchuan section of the Yellow River | River | YR1 | 38.11550 N | 106.22249 E |
|  |  |  | YR2 | 38.21749 N | 106.26732 E |
|  |  |  | YR3 | 38.30502 N | 106.34078 E |
|  |  |  | YR4 | 38.34488 N | 106.35314 E |
|  |  |  | YR5 | 38.38149 N | 106.42181 E |
|  |  |  | YR6 | 38.43745 N | 106.52206 E |
|  |  |  | YR7 | 38.51701 N | 106.54815 E |

**Table S2** Information on sampling sites for water samples in the basin

| Order | Family | Genus | Species | Common Name | Status in  China | Trophic  Levels | Migration Types | Feeding  Types |
| --- | --- | --- | --- | --- | --- | --- | --- | --- |
| Beloniformes | Adrianichthyidae | Oryzias | *Oryzias latipes* | Japanese  medaka | Native | Medium | Amphidromous | Omnivorous |
| Centrarchiformes | Centrarchidae | Micropterus | *Micropterus salmoides* | American black  bass | Introduced | High | Non-migratory | Carnivorous |
| Cichliformes | Cichlidae | Oreochromis | *Oreochromis mossambicus* | African mouthbrooder | Introduced | Low | Amphidromous | Planktivorous |
| Cypriniformes | Cyprinidae | Cyprinus | *Cyprinus carpio* | common carp | Native | Medium | Potamodromous | Carnivorous |
| Cypriniformes | Cyprinidae | Schizothorax | *Schizothorax sinensis* | Chinese Schizothorax | Native | Low | Potamodromous | Omnivorous |
| Cypriniformes | Danionidae | Danio | *Danio rerio* | zebrafish | Introduced | Medium | Non-migratory | Carnivorous |
| Cypriniformes | Gobionidae | Pseudorasbora | *Pseudorasbora parva* | topmouth gudgeon | Native | Medium | Non-migratory | Omnivorous |
| Cypriniformes | Xenocyprididae | Hypophthalmichthys | *Hypophthalmichthys nobilis* | bighead  carp | Endimic | Medium | Potamodromous | Omnivorous |
| Siluriformes | Siluridae | Silurus | *Silurus meridionalis* | Chinese  large-mouth catfish | Endimic | High | Non-migratory | Carnivorous |

**Notes:** Trophic levels were categorized as "low trophic" (trophic index ≤ 2.8), "medium trophic" (2.8 < trophic index < 3.5), and "high trophic" (trophic index ≥ 3.5). The above data were primarily sourced from the FishBase database.

**Table S3** Mitochondrial genome information of nine fish

| Species | GenBank  Accession | TaxID |
| --- | --- | --- |
| *Oryzias latipes* | NC_004387.1 | 8090 |
| *Micropterus salmoides* | NC_014686.1 | 489037 |
| *Oreochromis mossambicus* | DQ426661.1 | 8127 |
| *Cyprinus carpio* | NC_001606.1 | 7962 |
| *Schizothorax sinensis* | NC_056907.1 | 1080309 |
| *Danio rerio* | MF139226.1 | 7955 |
| *Pseudorasbora parva* | MT413335.1 | 51549 |
| *Hypophthalmichthys nobilis* | NC_010194.1 | 7695 |
| *Silurus meridionalis* | NC_014866.1 | 175797 |

**Table S4** Congeneric species considered during species-specific primer design for mitochondrial genome sequence alignment

| Target Species | Genus | Congeneric Species Considered | | Mitochondrial Region Selected |
| --- | --- | --- | --- | --- |
|  |  | Species | GenBank Accession |  |
| *Schizothorax*  *sinensis* | Schizothorax | *Schizothorax lissolabiata* | NC_027162.1 | ND1, ND2 |
|  |  | *Schizothorax dolichonema* | NC_023979.1 |  |
|  |  | *Schizothorax taliensis* | NC_037516.1 |  |
|  |  | *Schizothorax griseus* | NC_046462.1 |  |
|  |  | *Schizothorax prenanti* | NC_023829.1 |  |
|  |  | *Schizothorax grahami* | NC_029708.1 |  |
|  |  | *Schizothorax davidi* | NC_026205.1 |  |
| *Silurus meridionalis* | Silurus | *Silurus glanis* | NC_014261.1 | ND1, Cytb |
|  |  | *Silurus lanzhouensis* | NC_015650.1 |  |
|  |  | *Silurus asotus* | NC_015806.1 |  |
|  |  | *SIlurus soldatovi* | NC_022723 |  |
|  |  | *Silurus microdorsalis* | NC_028175 |  |
|  |  | *Silurus grahami* | NC_056098.1 |  |

**Table S5** Fish primer sequence information

| Species | Mitochondrial  Genome  Region | Primer  Name | Primer Sequence（5' - 3'） | Tm | Product  Length  （bp） | Resource |
| --- | --- | --- | --- | --- | --- | --- |
| *Oryzias*  *latipes* | ND5 | OlaND5 | F：TCTTTACTATAATCCTGGCAGTCCTTATC | 59.9 | 108 | (Tsuji et al., 2018) |
|  |  |  | R：CTGCTGCTAACTCTTTTTGTTGTTC | 58.8 |  |  |
|  | Cytb | Ola | F：TGCCGCCGCAACAGTT | 56.8 | 108 | (Hoshino et al., 2021) |
|  |  |  | R：GAAAAGTAAGGGTGGAAGGATACTT | 56.2 |  |  |
| *Micropterus*  *salmoides* | Cytb | Msa | F：GCCCACATTTGTCGTGATGTAA | 59.5 | 106 | (Jo et al., 2021) |
|  |  |  | R：AACCCCGGCCGATATG | 58.4 |  |  |
|  | 16s | MiSa_16S | F：ACATCCCGAAACAAAGGGCT | 56.3 | 143 | (Clusa & García-Vázquez, 2018) |
|  |  |  | R：AATTCTGTTCATTAGAGCGGAGG | 58.1 |  |  |
| *Oreochromis*  *mossambicus* | 16s | 16S_Oreo | F：CTTCAGACGCCAGAACAG | 54.8 | 164 | (Noble et al., n.d.) |
|  |  |  | R：GCTTGGAGTTGTAACTCTGG | 54.2 |  |  |
|  | 12s | 12S_Oreo | F：AGCTTACCCTGTGAAGGAAC | 54.4 | 102 |  |
|  |  |  | R：AGGGAATGTAGCCCATTTC | 51.7 |  |  |
| *Cyprinus*  *carpio* | COI | CYCA_COI | F：CCACTAATAATCGGAGCCCCA | 57.1 | 173 | (Hernandez et al., 2020) |
|  |  |  | R：GCTCCTGCGTGGGCTAAG | 59.0 |  |  |
|  | Cytb | CpCyB_496 | F：GGTGGGTTCTCAGTAGACAATGC | 58.1 | 78 | (Takahara et al., 2012) |
|  |  |  | R：GGCGGCAATAACAAATGGTAGT | 56.7 |  |  |
| *Schizothorax*  *sinensis* | ND1 | Ss_ND1_2 | F：CTTCGCGCTATTTTTCC | 51.1 | 246 | SelfDesign |
|  |  |  | R：ACGAAGGCTAGTGTGA | 53.8 |  |  |
|  | ND2 | Ss_ND2 | F：CAATCCTATTATCCAGCCTAG | 54.7 | 235 |  |
|  |  |  | R：CTTCACTCTCCTGTTGCTCA | 53.4 |  |  |
| *Danio rerio* | Cytb | Dre_cytb_V132 | F：CTTACGTGGGAGATACCCTAGTG | 57.9 | 132 | (Hirohara et al., 2021) |
|  |  |  | R：GCAAGTGTAGAATAACTATGGCGATG | 59.9 |  |  |
|  | COI | Dre_COI | F：GGTGCTTGAGCCGGAATAGT | 59.3 | 73 | (B. Zhao et al., 2021) |
|  |  |  | R：GTGCTCCTGGTTGGCTAAGT | 56.8 |  |  |
| *Pseudorasbora*  *parva* | COI | Ppar_CO1 | F：CCCTGACATAGCATTCCCC | 54.4 | 79 | (Clusa & García-Vázquez, 2018) |
|  |  |  | R：AGAGGCTAAGAGTAGGAGGA | 55.7 |  |  |
|  | COI | Pp_COI | F：GTGTTTCATCAATTCTAGGAGCAAT | 59.1 | 101 | (Manfrin et al., 2022) |
|  |  |  | R：AGCTCATACAAATAAGGGCGTTTGA | 63.8 |  |  |
| *Hypophthalmichthys nobilis* | COI | HYNO_COI_2 | F：TTAGGGGCAATTAACTTCATCACC | 61.8 | 124 | (Farrington et al., 2015) |
|  |  |  | R：GTAAGGATAGGAGAAGAAGTACGGCC | 62.9 |  |  |
|  | ND2 | BHTM1 | F：TAGACCTTCTAACAGGACTAATTC | 54.3 | 144 | (Farrington et al., 2015) |
|  |  |  | R：AATCCACCTCATCCTCCAAC | 53.5 |  |  |
| *Silurus meridionalis* | ND1 | Sm_ND1 | F：AGCCTTACTAATAACACATGTAG | 53.1 | 112 | Self Design |
|  |  |  | R：TAATTGTATGTATCCTAGTACTTTC | 51.3 |  |  |
|  | Cytb | Sm_Cytb1 | F：CCGCCGTACCATACATAG | 53.1 | 140 |  |
|  |  |  | R：GGCGTGGAGAAGTGTG | 51.7 |  |  |
|  | Cytb | Sm_Cytb2 | F：TCAAGCCAGAGTGATACTTC | 53.4 | 213 |  |
|  |  |  | R：CGATTCAGGTTAGGATTAGGA | 53.7 |  |  |

Notes：The detail of resource reference is shown in the end of this Supplementary Material.

**Table S6 BLASTN alignment results of cloned plasmid inserts against the NCBI GenBank database.**

| Species | GenBank  Accession | Primer  Name | Query Coverage  (%) | Percent Identity  (%) | E value |
| --- | --- | --- | --- | --- | --- |
| *Oryzias latipes* | NC_004387.1 | OlaND5 | 100 | 99.07 | 1×10^-46^ |
|  |  | Ola | 100 | 100 | 2.52×10^-45^ |
| *Micropterus salmoides* | NC_014686.1 | Msa | 100 | 100 | 3×10^-47^ |
|  |  | MiSa_16S | 100 | 99.30 | 1.59×10^-62^ |
| *Oreochromis mossambicus* | DQ426661.1 | 16S_Oreo | 100 | 100 | 1.79×10^-75^ |
|  |  | 12S_Oreo | 100 | 99.02 | 3×10^-43^ |
| *Cyprinus carpio* | NC_001606.1 | CYCA_COI | 100 | 100 | 2.49×10^-80^ |
|  |  | CpCyB_496 | 100 | 100 | 2.85×10^-29^ |
| *Schizothorax sinensis* | NC_056907.1 | Ss_ND1_2 | 100 | 100 | 9.03×10^-120^ |
|  |  | Ss_ND2 | 100 | 98.30 | 8×10^-112^ |
| *Danio rerio* | MF139226.1 | Dre_cytb_V132 | 100 | 99.24 | 1.34×10^-56^ |
|  |  | Dre_COI | 100 | 100 | 1.29×10^-26^ |
| *Pseudorasbora parva* | MT413335.1 | Ppar_CO1 | 100 | 100 | 8.36×10^-30^ |
|  |  | Pp_COI | 100 | 99.01 | 6.07×10^-47^ |
| *Hypophthalmichthys nobilis* | NC_010194.1 | HYNO_COI_2 | 100 | 99.31 | 5×10^-67^ |
|  |  | BHTM1 | 100 | 100 | 3×10^-106^ |
| *Silurus meridionalis* | NC_014866.1 | Sm_ND1 | 100 | 100 | 2×10^-50^ |
|  |  | Sm_Cytb1 | 100 | 100 | 7×10^-66^ |
|  |  | Sm_Cytb2 | 100 | 100 | 3×10^-106^ |

Note: Percent identity ≥ 98% was used as the threshold for species confirmation.

**Table S7** Information of 32 sampling sites for ddPCR experiments

| Regions | Sampling Sites | Primer | Fish Species |  |
| --- | --- | --- | --- | --- |
| Gan River | Up2 | CYCA_COI | *Cyprinus carpio* | |
|  | Up2 | Ss_ND1_2 | *Schizothorax sinensis* | |
|  | Up5 | CYCA_COI | *Cyprinus carpio* | |
|  | Up8 | Dre_COI | *Danio rerio* | |
|  | YZ | CYCA_COI | *Cyprinus carpio* | |
|  | YZ | Ss_ND1_2 | *Schizothorax sinensis* | |
|  | Down1 | CpCyB_496 | *Cyprinus carpio* | |
|  | Down1 | CYCA_COI | *Cyprinus carpio* | |
|  | Down1 | Ss_ND1_2 | *Schizothorax sinensis* | |
|  | Down2 | CYCA_COI | *Cyprinus carpio* | |
|  | Down2 | Dre_cytb_V132 | *Danio rerio* | |
|  | Down2 | OlaND5 | *Oryzias latipes* | |
|  | Down2 | Pp_COI | *Pseudorasbora parva* | |
|  | Down2 | Ppar_CO1 | *Pseudorasbora parva* | |
| Jinshui River Basin | GH1 | 12S_Oreo | *Oreochromis mossambicus* | |
|  | GH1 | CYCA_COI | *Cyprinus carpio* | |
|  | GH1 | HYNO_COI_2 | *Hypophthalmichthys nobilis* | |
|  | GH1 | MiSa_16S | *Micropterus salmoides* | |
|  | GH1 | Msa | *Micropterus salmoides* | |
|  | GH5 | BHTM1 | *Hypophthalmichthys nobilis* | |
|  | GH5 | CYCA_COI | *Cyprinus carpio* | |
|  | GH5 | Sm_Cytb1 | *Silurus meridionalis* | |
|  | GH8 | CYCA_COI | *Cyprinus carpio* | |
|  | GH9 | Ss_ND1_2 | *Schizothorax sinensis* | |
|  | FT1 | CpCyB_496 | *Cyprinus carpio* | |
|  | FT1 | CYCA_COI | *Cyprinus carpio* | |
|  | FT1 | Dre_cytb_V132 | *Danio rerio* | |
|  | FT1 | HYNO_COI_2 | *Hypophthalmichthys nobilis* | |
|  | FT1 | Msa | *Micropterus salmoides* | |
|  | FT6 | MiSa_16S | *Micropterus salmoides* | |
|  | FT10 | 16S_Oreo | *Oreochromis mossambicus* | |
|  | FT10 | BHTM1 | *Hypophthalmichthys nobilis* | |
|  | FT14 | 12S_Oreo | *Oreochromis mossambicus* | |
|  | FT14 | CYCA_COI | *Cyprinus carpio* | |
|  | FT14 | Ss_ND1_2 | *Schizothorax sinensis* | |
|  | FT14 | MiSa_16S | *Micropterus salmoides* | |
|  | FT14 | Sm_ND1 | *Silurus meridionalis* | |
|  | JS11 | CpCyB_496 | *Cyprinus carpio* | |
|  | JS11 | Ppar_CO1 | *Pseudorasbora parva* | |
|  | JS12 | Ss_ND1_2 | *Schizothorax sinensis* | |
| Lushui River | LS1 | CYCA_COI | *Cyprinus carpio* | |
|  | LS1 | Ss_ND1_2 | *Schizothorax sinensis* | |
|  | LS5 | CYCA_COI | *Cyprinus carpio* | |
|  | LS6 | CYCA_COI | *Cyprinus carpio* | |
|  | LS6 | Ss_ND1_2 | *Schizothorax sinensis* | |
|  | LS6 | Msa | *Micropterus salmoides* | |
|  | LS8 | CYCA_COI | *Cyprinus carpio* | |
|  | LS11 | CYCA_COI | *Cyprinus carpio* | |
| Ge Lake | GL1 | Sm_Cytb2 | *Silurus meridionalis* | |
|  | GL1 | Ola | *Oryzias latipes* | |
|  | GL41 | CYCA_COI | *Cyprinus carpio* | |
| Shengjin Lake | SJ12 | Ss_ND1_2 | *Schizothorax sinensis* | |
|  | SJ23 | 16S_Oreo | *Oreochromis mossambicus* | |
| Yinchuan section of the Yellow River | YR1 | Ss_ND2 | *Schizothorax sinensis* | |
|  | YR2 | CpCyB_496 | *Cyprinus carpio* | |
|  | YR2 | CYCA_COI | *Cyprinus carpio* | |
|  | YR3 | Ss_ND1_2 | *Schizothorax sinensis* | |
|  | YR3 | Ss_ND2 | *Schizothorax sinensis* | |
|  | YR4 | Ss_ND1_2 | *Schizothorax sinensis* | |
|  | YR4 | Ss_ND2 | *Schizothorax sinensis* | |
|  | YR5 | Ss_ND1_2 | *Schizothorax sinensis* | |
|  | YR5 | Ss_ND2 | *Schizothorax sinensis* | |
|  | YR6 | Ss_ND1_2 | *Schizothorax sinensis* | |
|  | YR6 | Ss_ND2 | *Schizothorax sinensis* | |
|  | YR7 | Ss_ND1_2 | *Schizothorax sinensis* | |
|  | YR7 | Ss_ND2 | *Schizothorax sinensis* | |

**Figure S1 - S3**

Figure S1 Representative field photographs of the sampling sites across the studied area. (A) Gan River. (B) Jinshui River Basin. (C) Lushui River. (D) Ge Lake. (E) Shengjin Lake. (F) Yinchuan section of the Yellow River.

Figure S2 Limit of Detection (LOD) and Limit of Quantification (LOQ) of HT-qPCR method

Figure S3 Performance Evaluation of Different eDNA Primers for Single Fish Species at Various Locations

**Reference**

Clusa, L., & García-Vázquez, E. (2018). A simple, rapid method for detecting seven common invasive fish species in Europe from environmental DNA. *Aquatic Conservation: Marine and Freshwater Ecosystems*, *28*(3), 619–629. https://doi.org/10.1002/aqc.2890

*Design of primers and probes for quantitative real-time PCR methods*. (n.d.). Retrieved August 28, 2024, from https://link.springer.com/protocol/10.1007/978-1-4939-2365-6_3

Farrington, H. L., Edwards, C. E., Guan, X., Carr, M. R., Baerwaldt, K., & Lance, R. F. (2015). Mitochondrial Genome Sequencing and Development of Genetic Markers for the Detection of DNA of Invasive Bighead and Silver Carp (Hypophthalmichthys nobilis and H. molitrix) in Environmental Water Samples from the United States. *PLoS ONE*, *10*(2), e0117803. https://doi.org/10.1371/journal.pone.0117803

Fuentes-Arderiu, X. (2002). *Quantifying the Capability of Detection of Measurement Procedures in Clinical Laboratory Sciences*. *40*(4), 425–425. https://doi.org/10.1515/CCLM.2002.070

Hernandez, C., Bougas, B., Perreault-Payette, A., Simard, A., Côté, G., & Bernatchez, L. (2020). 60 specific eDNA qPCR assays to detect invasive, threatened, and exploited freshwater vertebrates and invertebrates in Eastern Canada. *Environmental DNA*, *2*(3), 373–386. https://doi.org/10.1002/edn3.89

Hirohara, T., Tsuri, K., Miyagawa, K., Paine, R. T. R., & Yamanaka, H. (2021). The Application of PMA (Propidium Monoazide) to Different Target Sequence Lengths of Zebrafish eDNA: A New Approach Aimed Toward Improving Environmental DNA Ecology and Biological Surveillance. *Frontiers in Ecology and Evolution*, *9*, 632973. https://doi.org/10.3389/fevo.2021.632973

Hoshino, T., Nakao, R., Doi, H., & Minamoto, T. (2021). Simultaneous absolute quantification and sequencing of fish environmental DNA in a mesocosm by quantitative sequencing technique. *Scientific Reports*, *11*(1), 4372. https://doi.org/10.1038/s41598-021-83318-6

Jo, T., Ikeda, S., Fukuoka, A., Inagawa, T., Okitsu, J., Katano, I., Doi, H., Nakai, K., Ichiyanagi, H., & Minamoto, T. (2021). Utility of environmental DNA analysis for effective monitoring of invasive fish species in reservoirs. *Ecosphere*, *12*(6), e03643. https://doi.org/10.1002/ecs2.3643

Manfrin, C., Mirimin, L., Zanetti, M., Pizzul, E., Giulianini, P. G., & Pallavicini, A. (2022). Highly sensitive environmental DNA detection of topmouth gudgeon, Pseudorasbora parva: A comparison of qPCR and microfluidic qdPCR. *Biological Invasions*, *24*(7), 2121–2133. https://doi.org/10.1007/s10530-022-02761-2

Noble, T. H., Robson, H. L. A., Saunders, R. J., & Jerry, D. R. (n.d.). *The utility of eDNA as a tilapia surveillance tool*.

Qin, Q., Chen, L., Zhang, F., Xu, J., & Zeng, Y. (2024). Characterization of the complete mitochondrial genome of schizothorax kozlovi (cypriniformes, cyprinidae, schizothorax) and insights into the phylogenetic relationships of schizothorax. *Animals*, *14*(5), 721. https://doi.org/10.3390/ani14050721

Takahara, T., Minamoto, T., Yamanaka, H., Doi, H., & Kawabata, Z. (2012). Estimation of Fish Biomass Using Environmental DNA. *PLoS ONE*, *7*(4), e35868. https://doi.org/10.1371/journal.pone.0035868

Tsuji, S., Iguchi, Y., Shibata, N., Teramura, I., Kitagawa, T., & Yamanaka, H. (2018). Real-time multiplex PCR for simultaneous detection of multiple species from environmental DNA: An application on two Japanese medaka species. *Scientific Reports*, *8*(1), 9138. https://doi.org/10.1038/s41598-018-27434-w

Ye, J., Coulouris, G., Zaretskaya, I., Cutcutache, I., Rozen, S., & Madden, T. L. (2012). Primer-BLAST: A tool to design target-specific primers for polymerase chain reaction. *BMC Bioinformatics*, *13*(1), 134. https://doi.org/10.1186/1471-2105-13-134

Zhao, B., Van Bodegom, P. M., & Trimbos, K. (2021). The particle size distribution of environmental DNA varies with species and degradation. *Science of The Total Environment*, *797*, 149175. https://doi.org/10.1016/j.scitotenv.2021.149175

Zhao, Y., Su, J.-Q., Ye, J., Rensing, C., Tardif, S., Zhu, Y.-G., & Brandt, K. K. (2019). AsChip: A High-Throughput qPCR Chip for Comprehensive Profiling of Genes Linked to Microbial Cycling of Arsenic. *Environmental Science & Technology*, *53*(2), 798–807. https://doi.org/10.1021/acs.est.8b03798

Zhu, J., Huang, Q., Peng, X., Zhou, X., Gao, S., Li, Y., Luo, X., Zhao, Y., Rensing, C., Su, J., Cai, P., Liu, Y., Chen, W., Hao, X., & Huang, Q. (2022). MRG Chip: A High-Throughput qPCR-Based Tool for Assessment of the Heavy Metal(loid) Resistome. *Environmental Science & Technology*, *56*(15), 10656–10667. https://doi.org/10.1021/acs.est.2c00488
